# Alternative responses to rare selection events are differentially vulnerable to changes in the frequency, scope, and intensity of environmental extremes

**DOI:** 10.1002/ece3.5675

**Published:** 2019-09-27

**Authors:** Thomas R. Haaland, Carlos A. Botero

**Affiliations:** ^1^ Department of Biology Centre for Biodiversity Dynamics Norwegian University of Science and Technology Trondheim Norway; ^2^ Department of Evolutionary Biology and Environmental Studies University of Zürich Zürich Switzerland; ^3^ Department of Biology Washington University in Saint Louis St. Louis MO USA

**Keywords:** adaptation, bet‐hedging, climate change, evolutionary rescue, evolutionary simulation model, extreme weather events, life history

## Abstract

Extreme weather events are becoming more frequent, severe, and/or widespread as a consequence of anthropogenic climate change. While the economic and ecological implications of these changes have received considerable attention, the role of evolutionary processes in determining organismal responses to these critical challenges is currently unknown. Here we develop a novel theoretical framework that explores how alternative pathways for adaptation to rare selection events can influence population‐level vulnerabilities to future changes in the frequency, scope, and intensity of environmental extremes. We begin by showing that different life histories and trait expression profiles can shift the balance between additive and multiplicative properties of fitness accumulation, favoring different evolutionary responses to identical environmental phenomena. We then demonstrate that these different adaptive outcomes lead to predictable differences in population‐level vulnerabilities to rapid increases in the frequency, intensity, or scope of extreme weather events. Specifically, we show that when the primary mode of fitness accumulation is additive, evolution favors ignoring environmental extremes and lineages become highly vulnerable to extinction if the frequency or scope of extreme weather events suddenly increases. Conversely, when fitness accumulates primarily multiplicatively, evolution favors bet‐hedging phenotypes that cope well with historical extremes and are instead vulnerable to sudden increases in extreme event intensity. Our findings address a critical gap in our understanding of the potential consequences of rare selection events and provide a relatively simple rubric for assessing the vulnerabilities of any population of interest to changes in a wide variety of extreme environmental phenomena.

## Graphical abstract

Extreme weather events are becoming more frequent, severe, and widespread as a consequence of anthropogenic climate change, and while the economic and ecological implications of these changes have received considerable attention, the extent to which organisms may adapt to them is entirely unknown. We develop a novel theoretical framework based on the alternative ways in which fitness can accumulate within natural populations and their potential effects on adaptation to rare selection events and demonstrate how these processes leave populations differentially vulnerable when extreme events regimes change. These findings address a critical gap in our understanding of the potential consequences of climate change and allow us to generate testable predictions that can be applied to a variety of species and environmental phenomena.

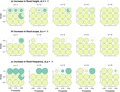

## 1 INTRODUCTION

Long‐term success in biology is sensitive to variation in performance (Frank & Slatkin, 1990; Lewontin & Cohen, 1969). In populations exposed to fluctuating environments, the trait values that confer high fitness under moderate conditions will occasionally be selected against if their bearers are negatively affected by environmental extremes. These recurring events facilitate the spread of alternative strategies that, while competitively inferior under moderate conditions are better able to cope with environmental extremes (Cohen, 1966; Simons, 2002). Such strategies, known as bet‐hedging, have attracted considerable attention in evolutionary biology and are known to occur in variety of systems with relatively high degrees of environmental uncertainty (Botero, Weissing, Wright, & Rubenstein, 2015; Simons, 2011; Starrfelt & Kokko, 2012; Xue, Sartori, & Leibler, 2019).

Theoretical accounts of bet‐hedging tend to emphasize the importance of maximizing geometric mean fitness in temporally variable environments because reproduction is an inherently multiplicative process (Dempster, 1955; Frank & Slatkin, 1990; Seger & Brockmann, 1987; Simons, 2011). The logic behind this premise is clearest when considering the case of a lineage that has historically performed well, but is suddenly unable to produce any offspring during a rare environmental extreme. The effect of that single value of zero fitness on the lineage's arithmetic mean fitness across generations is small, giving the false impression that the long‐term consequences of environmental extremes are relatively minimal. In contrast, a single generation with zero fitness drives the geometric mean fitness to zero, correctly highlighting that no matter how well a lineage may perform under moderate conditions, it simply has no expectation of persisting over the long‐term if it fails to reproduce during rare environmental extremes (Simons, 2002; Starrfelt & Kokko, 2012).

Predicting long‐term persistence is more complicated in spatially heterogeneous environments where different individuals are likely to experience different microhabitats. In such scenarios, geometric means often overestimate the importance of extreme conditions because not all trait carriers are exposed to a given environmental extreme, and hence do not experience the same fitness costs (see Jacquier, Kane, & Marcus, 2003). If the success that a trait value confers to unaffected carriers outweighs the losses it begets on affected ones, then the trait value can continue to be well represented in the population even if individual bearers are completely unable to deal with extreme conditions. Thus, spatial environmental heterogeneity can reduce the competitive edge of bet‐hedging strategies in variable environments and may drive populations to evolve phenotypes that simply maximize performance under typical conditions (Frank, 2011; Levins, 1962; Scheiner, 2014; Seger & Brockmann, 1987). A similar outcome is possible when reproduction depends on the prior accumulation of resources or when individuals exhibit multiple independent reproductive events in their lifetime. In those situations, even affected trait carriers can exhibit relatively high fitness if their lifetime reproductive output under moderate conditions outweighs the missed opportunities or costs accrued during environmental extremes. Arithmetic means are a better estimate of relative success in these situations and are therefore routinely used in fields such as behavioral ecology and game theory (Houston & McNamara, 1999; Parker & Maynard Smith, 1990).

**Figure 1:**
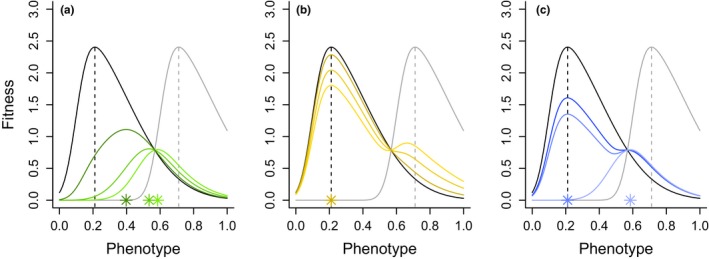
Fitness as a function of phenotype in fluctuating environments. In all panels, black and gray lines, respectively, depict fitness under moderate, *W*_M_ (*z*) and extreme conditions, *W*_E_ (*z*). Dashed vertical lines represent the maxima of *W*_M_ (*z*) and *W*_E_ (*z*). Colored lines depict long‐term fitness when fitness accumulation is assumed to be (a) strictly multiplicative or (b) strictly additive at different probabilities of experiencing extreme conditions (progressively darker lines depict *p* = .25, .15 and .05). (c) Long‐term fitness when fitness compounds both additively and multiplicatively, as defined by the scope of (i.e., proportion of individuals affected by) the extreme events, *s*. In this panel, progressively darker lines depict *s* = 1, 0.9 and 0.8. Colored stars at the bottom of each panel depict the maxima of the corresponding fitness functions. The fitness functions are described by skew‐Gaussian distributions with location parameters of 0.1 (moderate) and 0.6 (extreme), a shape parameter of 0.3, and a skew parameter of 5

The effect of shifting the balance between additive and multiplicative aspects of fitness accumulation can be further clarified by considering the shape of mean fitness functions under different accumulation scenarios. Consider a species that reproduces only once per lifetime and is exposed to environmental extremes with a small probability *p*. The expected response to rare selection events when fitness accumulation is strictly multiplicative, such as when environmental conditions affect the entire population at the same time (i.e., coarse environmental grain, Hedrick, Ginevan, & Ewing, 1976; Starrfelt & Kokko, 2012), can be estimated through a simple geometric mean,

(1) $${\bar{W}}_{G}\left(z\right)=W_{M}{\left(z\right)}^{\left(1-p\right)}\cdot W_{E}{\left(z\right)}^{p},$$

where *W*_M_ (*z*) and *W*_E_ (*z*) are the corresponding fitness functions of a nonplastic trait, *z*, under moderate and extreme conditions (Figure 1). Although the skewed fitness functions used in Figure 1 assume that phenotypes maximizing performance under moderate or extreme conditions perform poorly in the opposite environment, a variety of alternative fitness functions yield similar insights to the ones described below (Appendix A) and are therefore not further discussed. The optimal phenotype in ${\bar{W}}_{G}\left(z\right)$ (darker green stars) is increasingly similar to that of *W*_E_ (*z*) (gray dashed line) when environmental extremes are more frequent (Figure 1a). Thus, in this context environmental extremes favor the evolution of conservative phenotypes (i.e., bet‐hedging traits) that minimize variance in performance across all possible environments in spite of being conspicuously suboptimal under typical conditions (Levins, 1962; Simons & Johnston, 2003; Starrfelt & Kokko, 2012). While not explicitly modeled here, it is also possible that this scenario could drive the evolution of diversification bet‐hedging by producing two types of specialized offspring, a moderate‐condition‐specialist and an extreme‐condition‐specialist, with respective probabilities of (1−*p*) and *p* (i.e., effective proportional betting, Cohen, 1966; Donaldson‐Matasci, Lachmann, & Bergstrom, 2008; Graham, Smith, & Simons, 2014).

Consider now the opposite extreme, where fitness effects are strictly additive and response to rare selection events is estimated by the arithmetic mean,

(2) $${\bar{W}}_{A}\left(z\right)=\left(1-p\right)\cdot W_{M}\left(z\right)+p\cdot W_{E}\left(z\right).$$

As stated above, this arithmetic mean more appropriately captures the relative success of individuals that exhibit multiple independent breeding attempts either in time (i.e., within their lifetime) or space (i.e., breed multiply in spatially heterogeneous environments). Contrary to the fully multiplicative scenario, major phenotypic changes are unlikely to evolve in this case because the phenotype favored by ${\bar{W}}_{A}\left(z\right)$ is identical to that favored by *W*_M_ (*z*) as long as environmental extremes are rare (which is the case as long as *p* < .5, Figure 1b).

Intermediate cases between these extremes can be modeled, among other ways, by altering the scope *s* of extreme events, where *s* is defined as the proportion of a population that is affected by a given environmental extreme. In environments with both spatial and temporal heterogeneity, the mean fitness function therefore has both additive and multiplicative components,

(3) $${\bar{W}}_{\text{A,G}}\left(z\right)=W_{M}{\left(z\right)}^{\left(1-p\right)}\cdot {\left[\left(1-s\right)\cdot W_{M}\left(z\right)+s\cdot W_{E}\left(z\right)\right]}^{p},$$

and exhibits an intermediate shape between ${\bar{W}}_{A}\left(z\right)$ and ${\bar{W}}_{G}\left(z\right)$. When extreme events affect the entire population, ${\bar{W}}_{\text{A,G}}\left(z\right)$ reduces to ${\bar{W}}_{G}\left(z\right)$ and trait evolution is highly sensitive to performance failures (Figure 1c). As *s* decreases, contributions to fitness from unaffected trait carriers become more important during extreme events and the mean fitness function becomes more similar to ${\bar{W}}_{A}\left(z\right)$ (Figure 1c).

The above considerations suggest that evolutionary responses to environmental extremes are likely to depend on the relative importance of additive versus multiplicative aspects of fitness accumulation. Below, we test this hypothesis through an evolutionary simulation model that investigates population‐level responses to rare selection events in species that exhibit different life histories (i.e., different number of reproductive events per lifetime) and/or experience environments with different spatial grains (i.e., vary in the fraction of population that is typically affected by environmental extremes, Crowley, Ehlman, Korn, & Sih, 2016; Levins, 1962; Starrfelt & Kokko, 2012). We then use our simulation framework to investigate how these evolutionary responses to historical climate patterns may affect future vulnerability to climate change.

## 2 MODEL SETUP

To help motivate our model, we consider the case of a riparian nesting bird that is occasionally exposed to flooding (Starrfelt & Kokko, 2012). Although this particular phenotype and environmental extreme were chosen for convenience, we demonstrate below that this example typifies a general framework that can be easily applied to a wide variety of cases in which the focal phenotype interacts with a fluctuating environmental parameter to determine survival and/or reproductive success. In the case of riparian nesting, nesting lower in the reeds increases protection against aerial predators but also increases vulnerability to rising water levels if a flooding event occurs (Hunter, Nibbelink, & Cooper, 2016; Martin, 1995). Our model therefore assumes that the probability of nest predation (or the proportion of chicks that is typically taken by predators) increases linearly with nest height, *z*, and that flooding events destroy all nests below water level, *h*, such that an individual's reproductive success in a given breeding attempt is defined as

(4) $$W\left(z;h\right)=\left\{\begin{array}{cc} 1-z, & z\geq h\\ 0, & z<h \end{array}\right..$$

This function simplifies the dynamics of our toy model but retains the important quality that “moderate” (nonflood events) and “extreme” conditions (flooding events) favor divergent phenotypic optima. To shift the balance between additive and multiplicative aspects of fitness accumulation in our simulations, we altered the number of times, *n*, that a trait is used/expressed during a lifetime (i.e., the number of nesting attempts per individual), as well as the fraction of the population that is typically affected by environmental extremes (i.e., flooding scope, *s*). Every modeling scenario and parameter combination described in this section was replicated 100 times in order to assess consistency in evolutionary outcomes. Simulations were encoded and run in R (R Core Team, 2016), and code is available in Data S3.

### 2.1 Fitness and reproduction

Our baseline model assumes discrete, nonoverlapping generations, and an adult lifespan of 1 year (i.e., each population is entirely composed of offspring produced the previous year). Each replicate simulation was initialized with populations of 5,000 haploid, asexual individuals, whose nest height phenotypes were stochastically sampled from a uniform distribution between 0 and 1. Population sizes were allowed to fluctuate thereafter depending on the number of surviving offspring from the previous year. Flooding occurred with probability *p* per time step, and flood height was kept constant at *h* = 0.4.

During rare flooding events, each nest in the population had an independent probability *s* (determined by the scope of floods) of being affected. Thus, the lifetime reproductive success of individual *i* was computed as

(5) $$w_{i}=\sum_{k=1}^{n}c\cdot W\left(z_{i};h_{i,k}\right),$$

to the nearest integer, where *k* is an index of *n* nesting attempts, *c* is a constant representing a measure of population intrinsic rate of increase (maximum clutch size), and *h_i,k_* equals 0.4 with probability *s* in case of flooding, and 0 otherwise. Whenever necessary, the total number of offspring recruited to the next generation was capped at the carrying capacity parameter, *K* = 5,000, through stochastic removal of offspring (we assume no difference in competitive ability between offspring produced in different nesting attempts).

Each surviving offspring inherited the nest height phenotype from its parent with small probability of mutation, *m* = 0.001. In case of mutation, the offspring's phenotype was equal to the parental value plus a random deviate drawn from a normal distribution with mean zero and standard deviation *m.size* = 0.05. To prevent logical inconsistencies, mutated phenotypes were constrained between 0 and 1 (i.e., nesting cannot occur below ground or in the air above the reeds).

### 2.2 Climate change

To explore the potential effects of climate change on populations with different evolutionary backgrounds, we allowed populations to evolve under a given set of conditions for 2,000 generations and then simulated the rapid onset of an increase in either the height (*h*), scope (*s*), or frequency (*p*) of flooding events. Populations were subsequently followed for an additional 200 generations to quantify extinction probability, as well as any changes in phenotypic values and population dynamics. We note that this protocol assumes that the rate of climate change is faster than that of mutation, which appears to be the most common scenario reported in the empirical literature (Bailey et al., 2017; Visser, Both, & Lambrechts, 2004). Note that the timeframe chosen to assess responses to climate change was motivated by preliminary simulations, which showed that consistent extinction driven by environmental changes happened rapidly. A larger number of generations were specifically avoided in this case to minimize the confounding effect of variable background mortality rates.

### 2.3 Model variants

The findings we present here are based on extensive simulation under a variety of modeling scenarios and mechanistic assumptions. For simplicity, we focus our results and discussion on the findings of our simplest model variant, which broadly captures the biologically relevant patterns we discovered through this exercise. This simple model assumes that flood levels are invariant, that flooding and predation influence only nestling survival, and that mutations are rare and have relatively small effects (i.e., *m* = 0.001 and *m.size* = 0.05)—choices that best match the case of a continuous trait for which discrete jumps from one adaptive peak to another are unlikely. For completeness, additional variants with a variety of modeling assumptions are briefly described below and presented in Appendix B. These variants include models where flood height is allowed to vary over time; intrinsic population growth rate is small; single mutations are able to cross the fitness valley between low‐ and high‐nesting optima; flooding and predation affect both nestling and adult survival; and generations overlap.

## 3 RESULTS

### 3.1 Modes of fitness accumulation help determine phenotypic response to rare selection events

**Figure 2:**
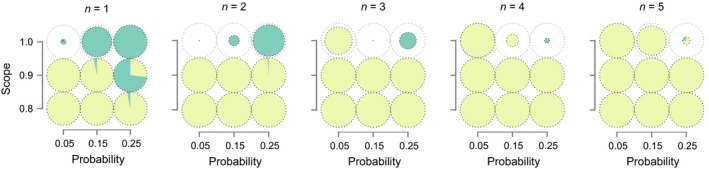
Evolved nest heights after 2,000 generations for populations with different numbers of reproductive events per lifetime, *n*. Each pie chart depicts the proportion of different evolutionary outcomes under a given combination of flooding probability and scope. Colors indicate whether populations evolved low‐nesting (yellow, $\bar{z}$ < 0.05), high‐nesting (green, $\bar{z}$ > *h*), or an intermediate mean phenotype (blue, 0.05 < $\bar{z}$ < *h*; note that this only occurs in a small slice of the top left pie in the *n* = 1 panel). The diameter of each pie depicts the proportion of all populations that survived until the end of the simulation

The evolutionary responses to rare selection events under different life histories, flooding scopes, and probabilities of flooding observed with our baseline model assumptions are summarized in Figure 2 (for additional model variants, see below and Appendix B). We use pie diameter in these plots to depict the proportion of surviving populations in the last generation (smaller pies indicate higher probabilities of extinction) and depict the mean frequency of each behavioral strategy in the last generation through the relative size of slices in each pie. Additionally, we provide the evolutionary trajectories of the individual populations that make up these pies in Data S1, and depict some representative examples graphically in Figure C.1 (Appendix C). Overall, our simulated populations evolved to either nest on the ground (hereafter “low nesting”) or just above flood level (“high nesting”). Observed variation in evolutionary outcomes was minimal both among replicates within parameter combinations (single pies in Figure 2, Figures B.1, B.6 and B.10 in Appendix B are only rarely multicolored), and among individuals within populations (i.e., genetic polymorphisms were generally unstable in this scenario, see Figure C.1 in Appendix C). As predicted by both our toy model (Figure 1, Figure A.1 in Appendix A) and previous theory (Levins, 1962; Seger & Brockmann, 1987; Starrfelt & Kokko, 2012), low nesting evolved in scenarios that emphasized the additive aspects of fitness accumulation (i.e., multiple clutches per lifetime and small flooding scopes—see also Figure C.5 in Appendix C for results with even smaller flooding scopes). The bet‐hedging high‐nesting strategy, on the other hand, evolved whenever multiplicative fitness accumulation was emphasized (i.e., frequent floods, large flooding scopes, and few clutches per lifetime).

Our simulations suggest that some patterns of environmental variability are simply difficult to adapt to. Specifically, when flooding events affected the entire population (*s* = 1), observed probabilities of extinction were often relatively high. For example, in scenarios with a high number of breeding attempts per season (*n* > 3), the likelihood of extinction (inversely depicted as pie diameters in Figure 2) increased with higher flood frequency. These populations had abundant opportunities to obtain fitness arithmetically and therefore evolved phenotypes that maximize fitness under moderate conditions. However, once low nesting became fixed, they were easily devastated if multiple flooding events occurred within a single season because such a string of environmental extremes was likely to affect every individual. Extinction was also relatively high when both flood frequency and the number of breeding attempts were low (*p* = .05 and *n* < 3). In this scenario, low‐nesting mutants typically emerged and outcompeted high‐nesters during the long interflood intervals (due to their lower susceptibility to predation) and populations became subsequently unable to maintain viable numbers when new flooding events occurred. The observation that bet‐hedging phenotypes are likely to be outcompeted by nonbet‐hedging alternatives when interflood intervals are long suggests that rare and unpredictable exposure to environmental extremes might favor either genetic and/or environmental canalization (Simons, 2002; Wagner, Booth, & Bagheri‐Chaichian, 1997), or lower rates of mutation (King & Masel, 2007).

### 3.2 Prior evolutionary history determines vulnerability to changes in extreme events

**Figure 3:**
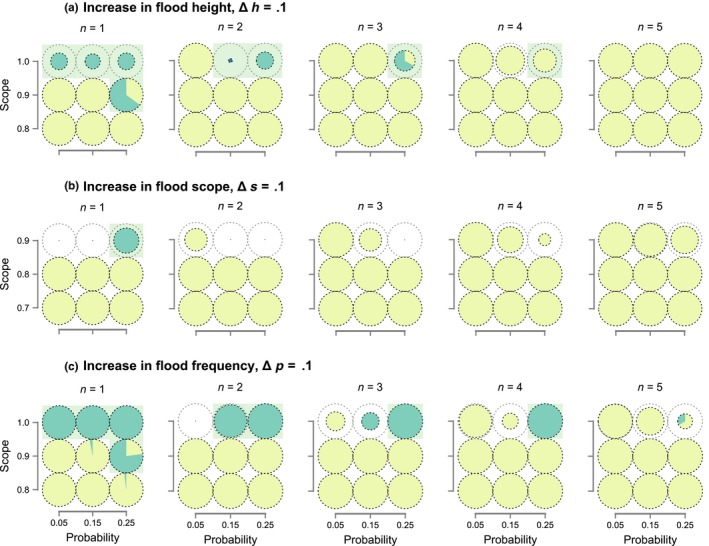
Effect of climate change on population viability and phenotypic evolution. In these two‐stage simulations, populations are allowed to evolve for 2,000 generations under the combinations of flooding probability and scope depicted on the *x*‐ and *y*‐axes and are subsequently subjected to a change in flooding regime. First row (a): Flood level is increased by 0.1. Second row (b): Scope of floods is increased by 0.1 (note different *y*‐axis values). Third row (c): Frequency of floods is increased by 0.1. The diameter of each pie represents the proportion of populations that survived for 200 generations after the change in flooding regime out of the pool of populations that had survived up until the change. Colors within the pie charts represent mean nesting phenotypes of surviving populations (low‐nesters in yellow, high‐nesters in green). For comparison, green shaded backgrounds indicate parameter combinations for which nesting above flood height evolved in more than 50% of the simulations without climate change (i.e., green pies in Figure 2)

Our findings support the notion that variation in life history and/or patterns of spatiotemporal environmental heterogeneity can result in selection for completely different evolutionary outcomes in response to identical climatic fluctuations (Scheiner, 2014). Additionally, they indicate that vulnerability to future changes in environmental cycles is likely to depend on the extent to which prior evolutionary history has promoted or not the evolution of phenotypes that can cope well with environmental extremes (Lawson, Vindenes, Bailey, & Pol, 2015; Olivieri, Tonnabel, Ronce, & Mignot, 2016). Although anthropogenic climate change is expected to result in longer, more severe, and/or more frequent extreme weather events (Hirabayashi et al., 2013; National Academy of Sciences, 2016; Stott, 2016; Trenberth, Fasullo, & Shepherd, 2015; Ummenhofer & Meehl, 2017), the extent to which populations may adapt to these changes is largely unknown (Donihue et al., 2018). To investigate this issue, we introduced a change in either flood height, flood scope, or probability of flooding at generation 2,000 and followed the resulting population dynamics and phenotypic evolution for an additional 200 generations. Figure 3, Figures B.2, B.4, B.5 and B.7 in Appendix B summarize our findings under a variety of parameter combinations and modeling assumptions. In these plots, we depict the proportions of phenotypes observed at generation 2,200 (pie slices) as well as the relative probability of survival, defined among all populations that survived to generation 2,000 as the proportion that had not gone extinct by generation 2,200 (pie diameter). The evolutionary trajectories of individual populations in these plots are also provided in Data S1, and representative examples of these dynamics are depicted graphically in Figure C.2 in Appendix C.

Our climate change simulations indicate that abrupt changes in the frequency or intensity of extreme weather events can dramatically reduce population viability, even in populations with a prior history of exposure to similar environmental extremes. Notably, vulnerability to different aspects of climate change varied depending on whether a population's prior evolutionary history had emphasized additive or multiplicative aspects of fitness accumulation. In evolutionary scenarios that emphasized the multiplicative aspects of fitness accumulation (green background in Figure 3, Figures B.2, B.4, B.5 and B.7 in Appendix B), all simulated populations exhibited conservative phenotypes by generation 2,000 and were subsequently unaffected by changes in the scope or frequency of similar environmental extremes (Figure 3b,c, Figures B.2b,c, B.5c and B.7b,c in Appendix B). However, these same populations were highly sensitive to changes in the height of floods because their nesting phenotype was not conservative enough to protect them against higher than expected water levels (Figure 3a, Figures B.2a, B.5a and B.7a in Appendix B), and because historically opposite forces of selection (i.e., flooding vs. predation) had largely depleted intrapopulation variation in nest height.

In simulations that emphasized additive aspects of fitness accumulation, we observed the exact opposite pattern of vulnerability to climate change. For example, these populations were completely unaffected by increases in flood level because they already experienced 100% flood‐related mortality prior to climate change. In contrast to populations in which fitness accumulates primarily multiplicatively, these populations were highly vulnerable to increased frequency or scope of environmental extremes (see pies without shaded backgrounds in top row of subplots, Figure 3b,c, Figure B2.b,c, B.5b,c and B.7b,c in Appendix B), because increasing either *p* or *s* reduced the fraction of nesting attempts that occurred under moderate conditions and shifted the balance between additive and multiplicative fitness accumulation. Specifically, fitness accumulation in these systems became increasingly multiplicative with more frequent or widespread floods, leading populations to experience an evolutionary tipping point (Botero et al., 2015), in which selection shifted from low to high nesting and the gradual accumulation of small‐effect mutations was not sufficient to avoid extinction.

Not all of the observed effects of climate change in our simulations were detrimental. In particular, the initially high baseline rates of extinction observed under very low flooding frequencies (Figure 2, Figure B.1 in Appendix B at *p* = .05 and *s* = 1) decreased dramatically after increasing flood frequency or flooding scope (Figure C.2c–d in Appendix C). This finding is consistent with the notion that population viability in fluctuating environments is highly dependent on the extent to which bet‐hedging strategies are able to resist invasion from nonbet‐hedging alternatives during periods of moderate conditions between consecutive environmental extremes (Simons, 2002). Specifically, the shorter interflood intervals related to higher flood frequencies enabled the maintenance of high‐nesting phenotypes in the population by reducing the likelihood that low‐nesting mutants would emerge and become fixed. More widespread flooding achieved a similar outcome by more thoroughly reducing the frequency of low nesters after each flooding event.

### 3.3 Other model assumptions change the details but not the general patterns

Altering the assumptions of our baseline model led to some predictable changes in response to selection that may be of use when considering particular species of interest. For example, genetic polymorphisms were not observed in our basic model but were common when generations overlapped, adult lifespan was long, and environmental extremes affected only nestling survival (Figures B.3a in Appendix B and C.3a in Appendix C). This finding is likely to be explained by an ecological “storage effect” (Chesson, 2000), where phenotypes that only cope well with moderate conditions are able to persist over long timescales because adult carriers are buffered from selection by rare environmental extremes. As expected, the presence of genetic polymorphisms significantly reduces population vulnerability to changes in frequency, scope, and intensity of environmental extremes because a section of the population will always possess the optimal postchange phenotype (Figure B.4 in Appendix B). Extinction risk was also reduced when single mutations allowed crossing the fitness valley between low‐ and high‐nesting optima (Figures B.8 and B.9 in Appendix B). In contrast, simulations with smaller intrinsic population growth rates (*c* = 2, rather than *c* = 5 as in the baseline model) predictably exhibited increased levels of extinction (Figures B.6 and B.9 in Appendix B) and stronger vulnerabilities to climate change (Figure B.7 in Appendix B). Allowing flood levels to vary over time yielded similar evolutionary outcomes as our basic model (i.e., the evolution of either high‐ or low‐nesting strategies, Figure B.10 in Appendix B), except that it forced high‐nesting lineages to add some “insurance” to their phenotype by nesting a little higher than their counterparts in models with invariant flood levels (Figure C.4 in Appendix C). Higher nesting phenotypes also incurred in higher predation levels and therefore showed slightly higher extinction risks. Finally, allowing flooding and predation to affect both nestling and adult survival lowered the expected number of breeding opportunities in a lifetime, and thus increased the multiplicative component of fitness accumulation. As a result, the number of populations that evolved bet‐hedging (high‐nesting) phenotypes increased (Figures B.1, B.3b, B.8b and B.9b in Appendix B), and populations that evolved under a wider range of conditions became vulnerable to changes in the intensity, but not the scope or frequency of environmental extremes (Figures B.2 and B.5 in Appendix B).

## 4 DISCUSSION

Our findings address fundamental outstanding questions with regards to the effect of rare selection events on evolutionary processes (Grant et al., 2017) and indicate that evolutionary parameters can shape vulnerability to climatic oscillations in somewhat unexpected ways. Our results challenge the previously suggested idea that species that have been exposed to more variable environments are better suited to cope with climate change and demonstrate instead that vulnerability is likely to depend on the interaction between life‐history and spatiotemporal variation (Lawson et al., 2015; McLean, Lawson, Leech, & Pol, 2016; Scheiner, 2014). First, we have shown that if a sufficient fraction of individuals in a population (or number of reproductive events in a lifetime) is able to escape the negative consequences of extreme events, then populations will typically evolve phenotypes that maximize fitness under moderate conditions (i.e., their phenotypes will ignore the possibility of exposure to environmental extremes) and will subsequently become most vulnerable to changes in the frequency or scope of environmental extremes. Conversely, we have shown that when escaping these consequences is difficult, populations will typically evolve bet‐hedging phenotypes and will instead become vulnerable to increases in the intensity of extreme events. We have also presented evidence consistent with the notion that factors that speed up trait evolution (e.g., easier mutational transition from one adaptive peak to another or higher mutation rates) are generally likely to hinder rather than favor adaptation to rare selection events because they tend to facilitate the competitive elimination of bet‐hedging strategies during periods of normality in between environmental extremes (King & Masel, 2007; Libby & Ratcliff, 2019; Simons, 2002). In practical terms, these findings suggest that an important first step for predicting the consequences of ongoing changes in the patterns of extreme weather is to identify whether life‐history strategies and prior exposure to environmental extremes have driven a species to evolve a phenotype that involves some fitness reduction under moderate conditions but enables its survival under environmental extremes (i.e., a bet‐hedging strategy). This relatively simple assessment, in consideration of the set of modeling assumptions that best matches the species of interest, should provide basic insights into potential vulnerabilities to climate change and should facilitate the identification of conservation priorities and the implementation of more effective management actions.

The simple framework we have outlined here can be easily extended to a variety of other natural systems and environmental phenomena. In addition to flooding, environmental extremes like wildfires, heatwaves, droughts, cold spells, tornadoes, and hurricanes are also increasing in intensity, scope, and/or frequency as a consequence of anthropogenic action (National Academy of Sciences, 2016; Trenberth et al., 2015; Ummenhofer & Meehl, 2017). To illustrate how our findings can also inform us about the potential consequences of those changes, we now use our findings to derive predictions for increases in the frequency, duration and geographic distribution of heatwaves (Ummenhofer & Meehl, 2017). Our model indicates that geographic regions in which heatwaves used to be rare and patchy are likely to host primarily species that do not exhibit conspicuous phenotypic adaptations to extreme heat (see Figure 2). Thus, the biggest threats of extinction in these areas will be more frequent and/or widespread heatwaves, and the species of highest concern will be endemics or other species whose small geographic distribution makes them more likely to have a large scope value (*s*) during environmental extremes (IUCN, 2012). Conversely, areas in which heatwaves were historically more common and widespread can be expected to host species that already exhibit adaptations for extreme heat and these species are likely to be more vulnerable to hotter than to longer or more widespread heatwaves.

The evolutionary dynamics highlighted in our model can also be useful for identifying potential threats of extinction in species of concern, and for designing more effective conservation actions. For example, two populations of the endemic Turks and Caicos anole, *Anolis scriptus*, were recently shown to have evolved larger toepads and shorter limb lengths (i.e., traits that increase these lizards' ability to cling to branches during strong winds) after two record‐breaking hurricanes (Donihue et al., 2018). These populations also exhibited a reduction in trait variance, suggesting that natural selection had favored individuals that were less likely to be blown away by hurricanes. Because Caribbean island anoles have substantially larger toepads than their mainland congeners despite the increased energetic costs associated with this trait (Donihue et al., 2018), we conclude that these island anoles have evolved conservative bet‐hedging phenotypes in response to occasional hurricane‐strength winds. Our results therefore suggest that while the Turks and Caicos anole (like the high‐nesting birds in our example, Figure 3) is likely to be unaffected by increasing hurricane frequencies, it may nevertheless face a significant threat of extinction if future hurricanes become significantly more intense (National Oceanic & Atmospheric Administration, 2018). Thus, the framework we have developed here suggests that a possible solution to this problem could be to reduce the scope of future hurricanes, for example, by providing wind refuges across the island that allow parts of the population to escape winds of very high intensity. While this simple conservation action is unlikely to completely shift the balance from mainly multiplicative to strictly additive fitness accumulation in this system, it may nevertheless enable the short‐term persistence of this species, allowing it to accumulate sufficient mutational changes to meet the new demands of its altered habitat.

Our results also offer a potential explanation to why many shorebirds that nest close to the shoreline have so far been unable to adjust their nesting behavior and are experiencing significant population declines in response to sea‐level rise (Bailey et al., 2017). Specifically, our model suggests that opposing selection from water levels and either habitat gradients or increased exposure to land predators may have favored a narrow range of nesting distances from the shoreline that is no longer adaptive under current conditions. If fast rates of trait evolution are detrimental for adaptation to fluctuating environments (as is apparent from Figure 3a, Figures B.2a, B.5a and B.7a in Appendix B), then it is also possible that the nesting behavior of these shorebirds has experienced selection for environmental canalization and that these populations are currently limited in their capacity to evolve a more appropriate nesting distance. Thus, our findings indicate that in this case, conservation may be difficult without explicit and sustained human action.

The ideas discussed here may also be useful for predicting the potential interactive effects of different aspects of climate change and anthropogenic action (Castorani, Reed, & Miller, 2018; Miller, Roxburgh, & Shea, 2011). For example, fire suppression practices have not only decreased the frequency of wildfires in large areas of the world, but have also intensified the fires that actually occur due to the increased buildup of fuel (Miller, Safford, Crimmins, & Thode, 2009; Scott, Bowman, Bond, Pyne, & Alexander, 2014; Steel, Safford, & Viers, 2015). Our model suggests that these changes represent a double threat to fire‐prone ecosystems because historical fire regimes in these regions are known to have promoted the evolution of bet‐hedging strategies, in which organisms sacrifice some short‐term fitness advantage (e.g., growth rate, competitive ability) in order to invest in costly traits that defend them against fires (such as thicker barks or a higher proportion of underground biomass). Our model therefore suggests that while these species are unlikely to be affected by more frequent or widespread fires, their persistence will be threatened by more intense fires. Furthermore, these threats of extinction are likely to be accompanied by an increased likelihood of invasion by less fire‐resistant but faster‐growing competitors as a consequence of longer intervals between fires (Ramage, O'Hara, & Caldwell, 2010; Tonnabel et al., 2012).

In conclusion, we have shown that the balance between arithmetic and multiplicative aspects of fitness accumulation largely determines how systems respond to selection from rare environmental extremes and shapes patterns of future vulnerability to climate change. These basic insights enable the formulation of general predictions on the likely consequences of a wide variety of environmental changes that are currently underway and represent an important advancement for the design and implementation of effective ecosystem management practices and conservation actions.

## CONFLICT OF INTEREST

None declared.

## AUTHOR CONTRIBUTIONS

Both authors developed the ideas and modeling framework. TRH ran the simulations. Both authors analyzed the results and wrote the manuscript.

## Data Availability

Computer code and all results are provided as electronic [Link], [Link], [Link].
