# Supplementary material for: Alternative responses to rare selection events are differentially vulnerable to changes in the frequency, scope, and intensity of environmental extremes
Source: Ecol Evol. 2019 Sep 27;9(20):11752–61. doi: 10.1002/ece3.5675 (PMC6822052; doi:10.1002/ece3.5675)
Supplement: Supplementary file 1 [file ECE3-9-11752-s001.docx]

Appendix A: Alternative fitness functions


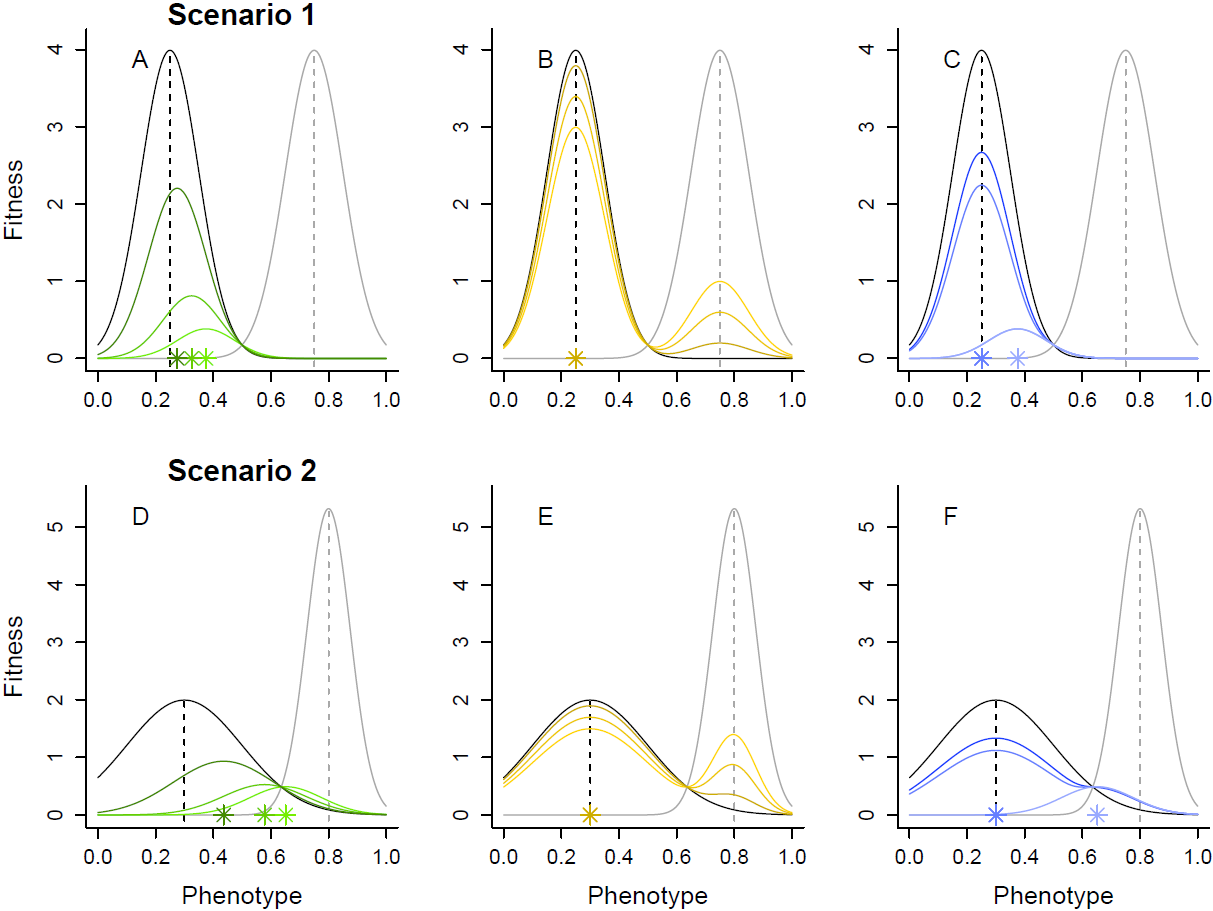


**Figure A.1:** Effects of different fitness functions on whether arithmetic and geometric fitness accumulation favors diverging phenotypic optima. In all panels, black and gray lines respectively depict fitness under moderate, *W_M_*(*z*), and extreme, *W_E_*(*z*), conditions. Dashed lines represent the maxima of *W_M_*(*z*) and *W_E_*(*z*). Scenario 1 (top row): Fitness functions are symmetrical Gaussian distributions with a mean of 0.25 (moderate) and 0.75 (extreme), and standard deviation of 0.1. Scenario 2 (bottom row): Fitness functions are symmetrical Gaussian distributions with different widths, means are 0.3 (moderate) and 0.8 (extreme), and standard deviations are 0.2 (moderate) and 0.075 (extreme). As in Figure 1 in the main text, colored lines depict long-term fitness when fitness accumulation is assumed to be strictly geometric (A, D) or strictly arithmetic (B, E) at different probabilities of extreme conditions (progressively darker lines depict *P* = 0.25, 0.15 and 0.05). (C, F) Long-term fitness when fitness accumulates both arithmetically and geometrically, as defined by the proportion of individuals affected by the extreme events, *s*. In these panels, progressively darker lines depict *s* = 1, 0.9, and 0.8. Colored stars at the bottom of each panel depict the maxima of the corresponding fitness functions. These plots demonstrate that our expectation that different types of fitness accumulation producing divergent phenotypic optima is likely to hold for a wide variety of fitness functions, but that the divergence is greatest when the capacity to cope with non-optimal environments is lower for phenotypes adapted to the moderate condition than for those adapted to the extreme conditions (c.f. larger distance between stars in scenario 2 than scenario 1).

Appendix B: Additional simulation model scenarios

Fitness costs apply to both adult and offspring survival: In this scenario, fitness values accrued from floods and predation (*W*(*z*; *h*) values, eq. 4) are assumed to affect adults as well as offspring. Specifically, a female with nesting phenotype *z* will have probability *W*(*z*; *h*) (which ranges between 0 and 1) of surviving, which of course depends on whether or not a flood occurs. If she survives, her reproductive success is (as in eq. 5) *w* = *c* * *W*(*z*; *h*). If she dies, she does not succeed in raising any offspring (i.e. she dies before laying the clutch, or the offspring die due to lack of parental care), but offspring fledged from any previous broods are assumed to survive, and can potentially be recruited to the adult population. The total reproductive success of a female *i* who if killed in time step *k* (*k* > 1) is thus $\sum_{j=1}^{k-1} w_{i,j}$. All other aspects of the model are similar as for that described elsewhere. We show results for this model in figures B.1 (no climate change, corresponding to figure 2 in main text) and B.2 (with climate change, corresponding to figure 3), and implement this model variant in several of the other scenarios as well, including those where generations overlap (figures B.3b, S10) and where mutations switch directly between the high- and low-nesting phenotypes (figures B.7b, B.8b).

Overlapping generations and longer lifespans: To examine the generality of the results in our model with discrete generations, we constructed a model variant where females can live for multiple years and offspring from multiple generations are allowed to overlap. For this purpose, we set *α* as the baseline between-year mortality, such that the expected lifespan (and also expected number of breeding opportunities per lifetime) becomes 1/α (0 < *α* ≤ 1; at *α* = 1 there is no adult survival between years and the model is identical to the main model of non-overlapping generations and one brood per year).

In this model variant, the simulation algorithm is identical to the one described above, except for the fact that adult survival now sets the limit to how many offspring can be recruited into the population any given generation. Specifically, the number of available ‘slots’ for offspring in this new model variant is determined as the carrying capacity *K* minus the number of surviving adults, *N*_adult_. Thus, the number of offspring recruited into any given generation, *N*_juv_, is either the total number of offspring produced the generation prior, Σ*W* * *c*, or *K* – *N*_adult_, whichever is smaller. In practical terms, this means that *N*_adult_ + *N*_juv_ is always smaller or equal to *K*. Offspring recruitment was then implemented by stochastically sampling *N_juv_* from the total offspring pool, which was generated using the same methods we describe in the main model variant. As above, these genotypes were then allowed to mutate given *m* = 0.001 and *m.size* = 0.05 (other model parameter values were also identical to those used in the main model variant).

Figures B.3 (without climate change) and B.4 and B.5 (with climate change) depict simulation outcomes in modeling instances with overlapping generations model that directly correspond to Figures 2 and B.1 (without climate change) and figures 3 and B.2 (with climate change) for the discrete generations model. In Figure C.3a-c, evolutionary trajectories (see Appendix C) of population means and variances are plotted for individual replicate simulations in the overlapping generations model.

Mutations switch phenotypes directly between discrete optima: Here we test the potential effects of our assumptions about genetic architecture on the observed simulation outcomes. Rather than assuming that *z* is a continuous trait with two discrete optima, where mutations add small random deviates to the parental trait value (see main text, Model setup 🡪 Fitness and reproduction), we now assume that *z* is entirely discrete, producing either a low (*z* = 0) or high (*z* = 0.4) nesting phenotype. If a mutation occurs (which has the same probability *m* = 0.001 as otherwise), the parental phenotype switches to the other phenotype without error. We show results for this model in Figures B.8 (with high population intrinsic rate of increase, *c*) and B.9 (low *c*), both of which show results for both types of fitness effects (floods and predation apply either only to offspring survival, or to both adult and offspring survival).

Variable flood heights: All scenarios until now have assumed that the height of floods *h* is constant (but changes to a higher value after 2000 generations in climate change simulations). In this model variant we investigate the effect of adding variability to flood heights across different flooding events. In these simulations, when a flood occurs its height is drawn stochastically from a Gaussian distribution around *h* with standard deviation *h*/10. The flood then affects a proportion *s* (flooding scope) of the current nests in the population, so that under a given flood, all nests experience the same flood height. The next time a flood occurs, which may be a later year, or during a later breeding cycle in the same year, a different flood height is drawn. We show results from these simulations in Figure B.10 as pie charts showing mean evolutionary outcomes across all parameter combinations, and in Figure C.4 as individual evolutionary trajectories (see Appendix C) compared with those for constant flood heights for certain representative parameter combinations.


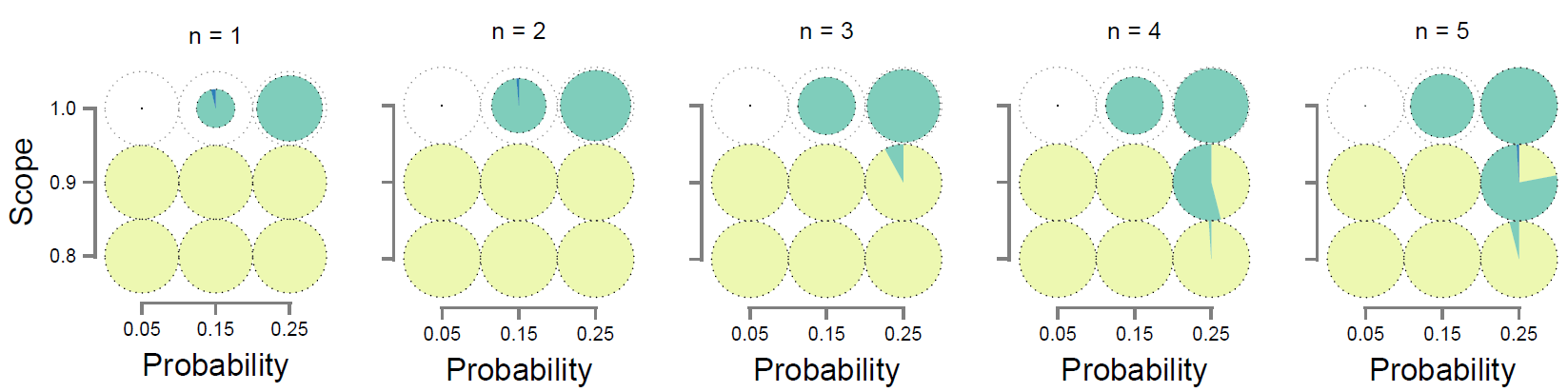


**Figure B.1.** Evolved nest heights after 2000 generations for populations with different numbers of reproductive events per lifetime, *n*. Each pie chart depicts the proportion of different evolutionary outcomes under the combination of flooding probability and scope depicted on the x- and y-axes. Fitness costs are assumed here to affect adult survival as well as reproductive success. Colors indicate whether populations evolved, on average, low-nesting (yellow, $\bar{z}$ < 0.05), high-nesting (green, $\bar{z}$ > *h*), or an intermediate phenotype (blue, 0.05 ≤ $\bar{z}$ ≤ *h*). The diameter of each pie depicts the proportion of populations that survived until the end of the simulation. 100 replicate simulations were run for each flooding scenario; the grid points with no pie chart indicate that no populations survived until the end of the simulation.


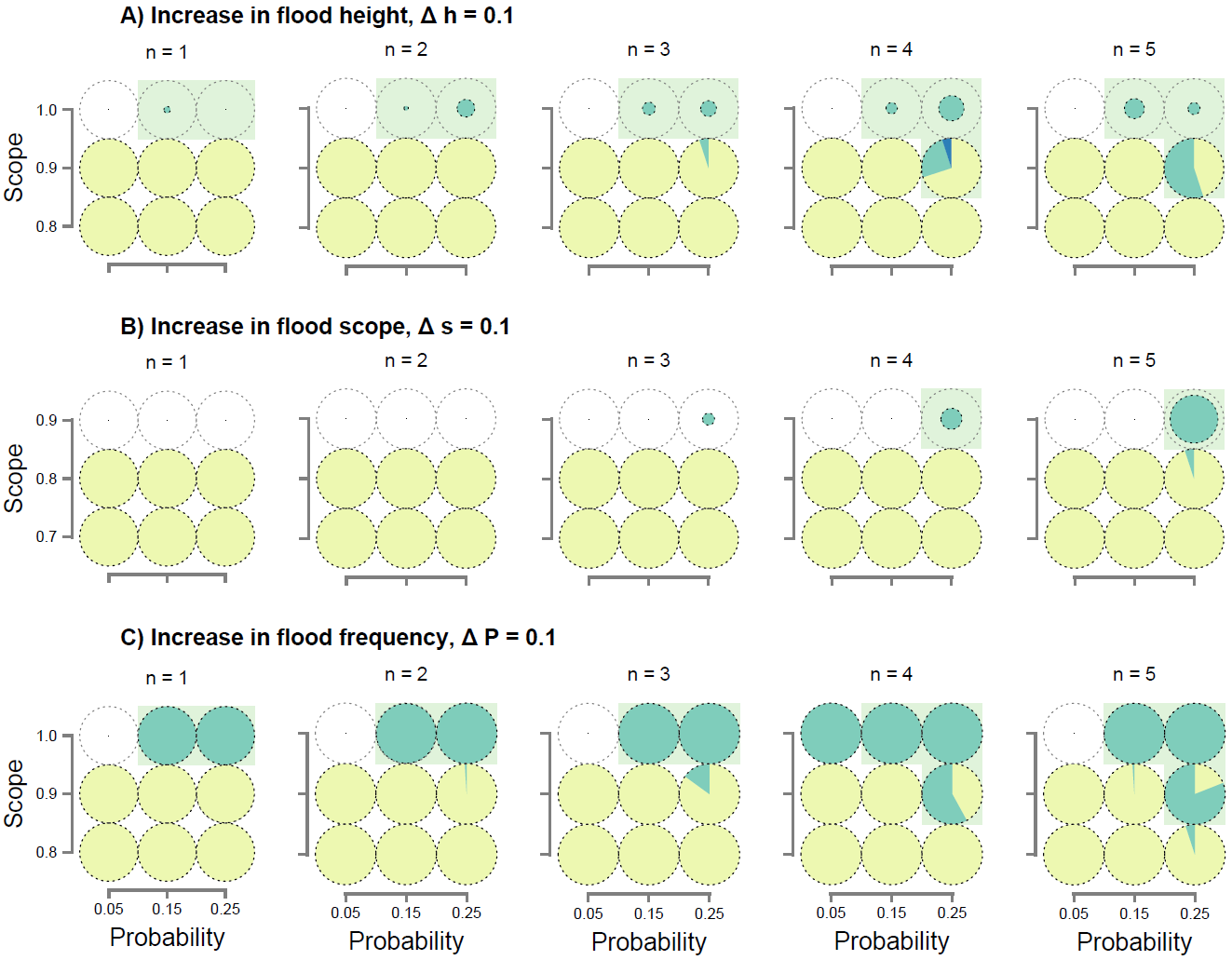


**Figure B.2.** Effect of climate change on population viability and phenotypic evolution when fitness costs are assumed to affect adult survival as well as reproductive success. In these two-stage simulations, populations first evolve for 2,000 generations under the combinations of flooding probability and scope depicted on the x- and y-axes, before a change in flooding regime is introduced. First row (A): Height of floods (when they occur) increases by 0.1. Second row (B): Scope of floods increases by 0.1 (note different y-axis). Third row (C): Frequency of floods increases by 0.1. The diameter of each pie represents the proportion of populations that survived for 200 generations after the change in flooding regime out of the pool of populations that had survived up until the change. Colors within the pie charts represent mean nesting phenotypes of surviving populations as in Figure 2. Light green background shading indicates parameter combinations for which nesting above flood height evolved in more than 50% of the corresponding simulations without climate change (i.e. locations in parameter space where pies were predominantly green in Figure B.1).

**
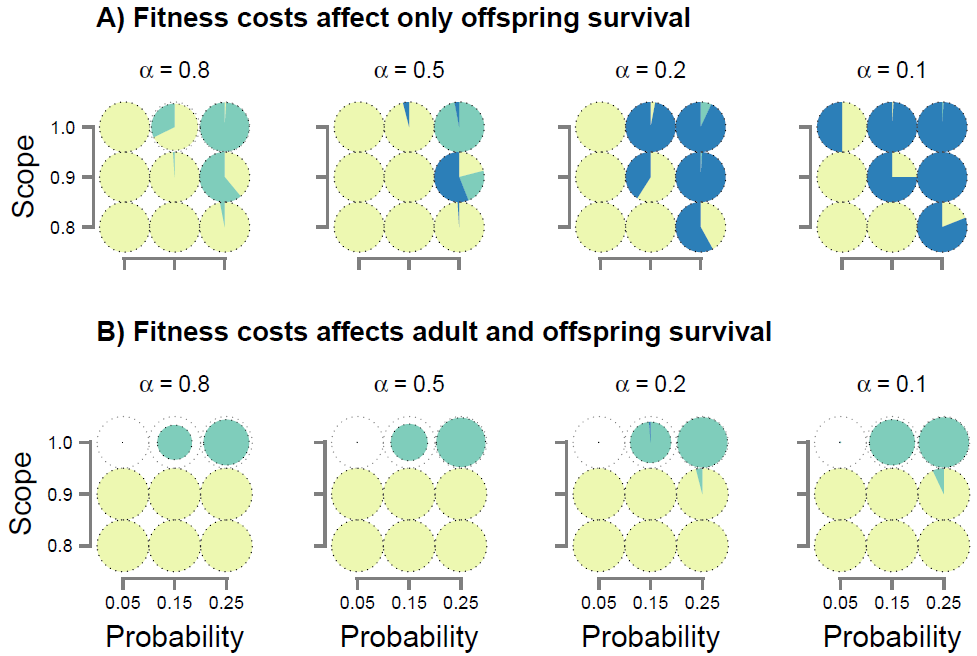
Figure B.3.** Evolved nest heights after 2000 generations for populations with overlapping generations. Rows indicate the target of fitness costs: In the top row (A), fitness costs affect only reproductive success, whereas in the bottom row (B) it affects adult survival as well as reproductive success. The top row is thus comparable with Figure 2 in the main text, and the bottom row with Figure B.1. Columns indicate between-year mortality *α*; from left to right, *α* = {0.8, 0.5, 0.2, 0.1}, which leads to expected lifespan of respectively 1.25, 2, 5 and 10 years. The diameter of each pie depicts the proportion of populations that survived until the end of the simulation. Colors indicate whether populations evolved low-nesting (yellow, $\bar{z}$ < 0.05), high-nesting (green, $\bar{z}$ > *h*), or were polymorphic (blue, 0.05 ≤ $\bar{z}$ ≤ *h*). 100 replicate simulations were run for each flooding scenario; the grid points with no pie chart indicate that no populations survived until the end of the simulation.  **
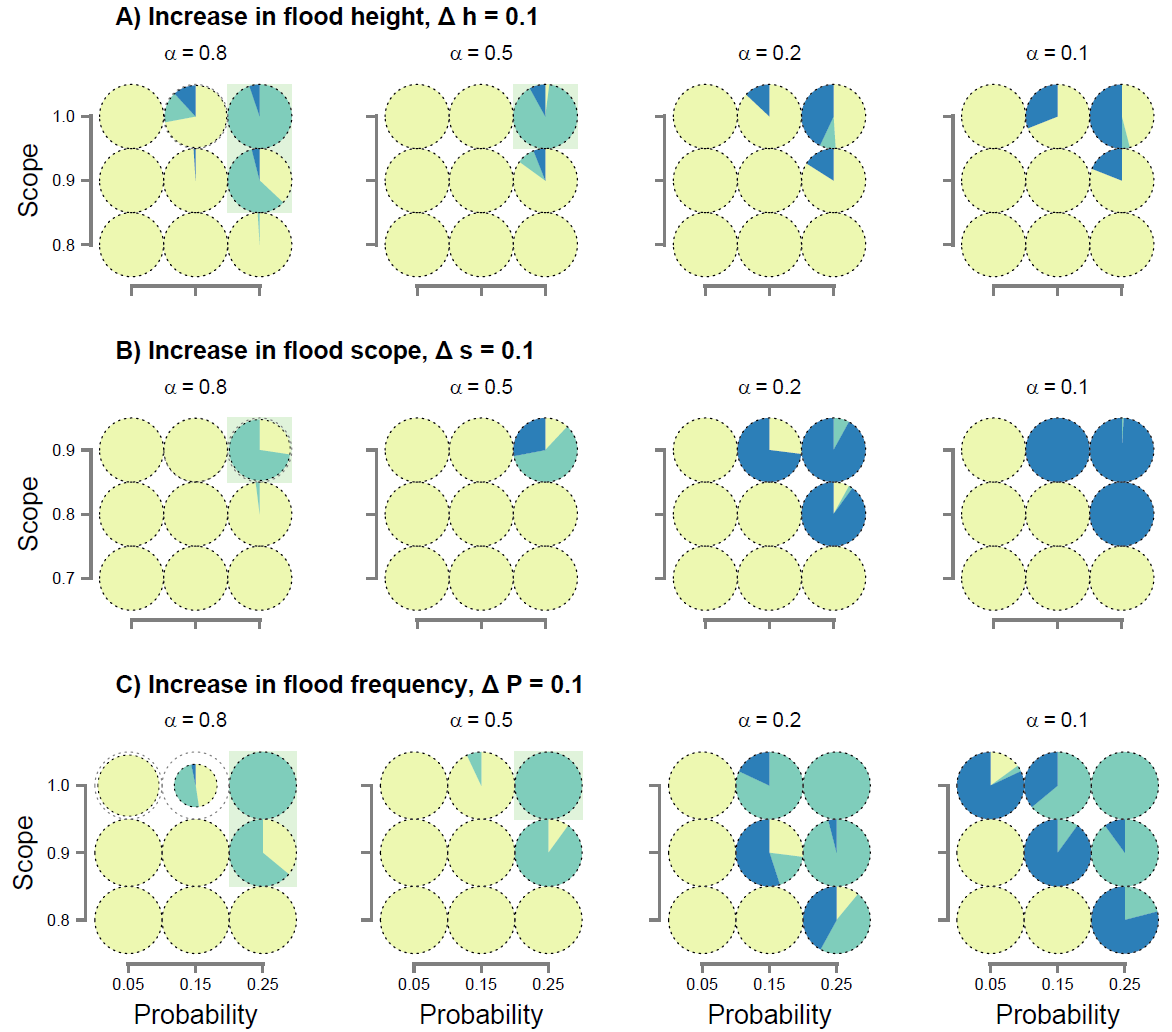
**

**Figure B.4.** Effect of climate change on population viability and phenotypic evolution when fitness costs affect only reproductive success, in the overlapping generations model. In these two-stage simulations, populations first evolve for 2,000 generations under the combinations of flooding probability and scope depicted on the x- and y-axes, before a change in flooding regime is introduced. First row (A): Flood level is increased by 0.1. Second row (B): Scope of floods is increased by 0.1 (note different y-axis values). Third row (C): Frequency of floods is increased by 0.1. The diameter of each pie represents the proportion of populations that survived for 200 generations after the change in flooding regime out of the pool of populations that had survived up until the change. Colors within the pie charts represent mean nesting phenotypes of surviving pie charts. Green shaded backgrounds indicate parameter combinations for which nesting above flood height evolved in more than 50% of the simulations without climate change (i.e. green pies in Figure B.3a).

**
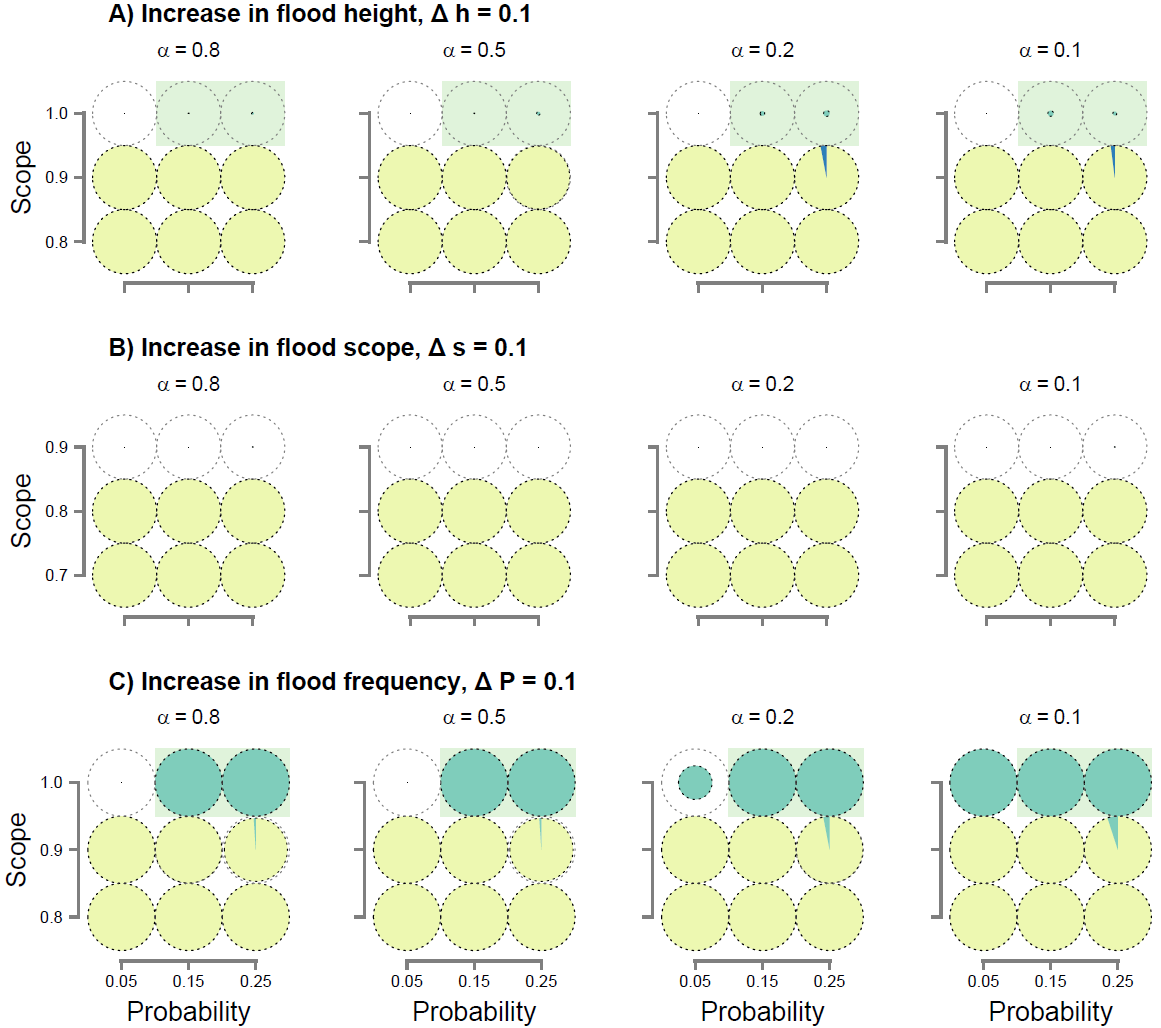
**

**Figure B.5.** Effect of climate change on population viability and phenotypic evolution when fitness costs affect adult survival as well as reproductive success, in the overlapping generations model. In these two-stage simulations, populations first evolve for 2,000 generations under the combinations of flooding probability and scope depicted on the x- and y-axes, before a change in flooding regime is introduced. First row (A): Flood level is increased by 0.1. Second row (B): Scope of floods is increased by 0.1 (note different y-axis values). Third row (C): Frequency of floods is increased by 0.1. The diameter of each pie represents the proportion of populations that survived for 200 generations after the change in flooding regime out of the pool of populations that had survived up until the change. Colors within the pie charts represent mean nesting phenotypes of surviving pie charts. Green shaded backgrounds indicate parameter combinations for which nesting above flood height evolved in more than 50% of the simulations without climate change (i.e. green pies in Figure B.3b).


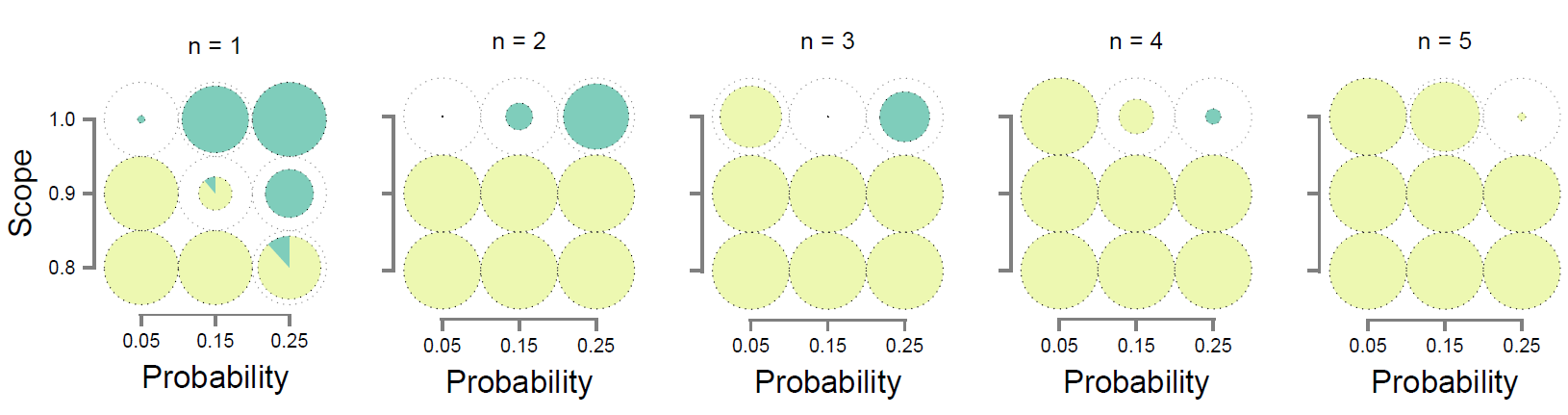


**Figure B.6.** Evolved nest heights after 2000 generations for populations with different numbers of reproductive events per lifetime, *n*, when parameter *c* (“clutch size”) is set to 2. Each pie chart depicts the proportion of different evolutionary outcomes under the combination of flooding probability and scope depicted on the x- and y-axes. Fitness costs affect only reproductive success. The diameter of each pie depicts the proportion of populations that survived until the end of the simulation. 20 replicate simulations were run for each flooding scenario; the grid points with no pie chart indicate that no populations survived until the end of the simulation.


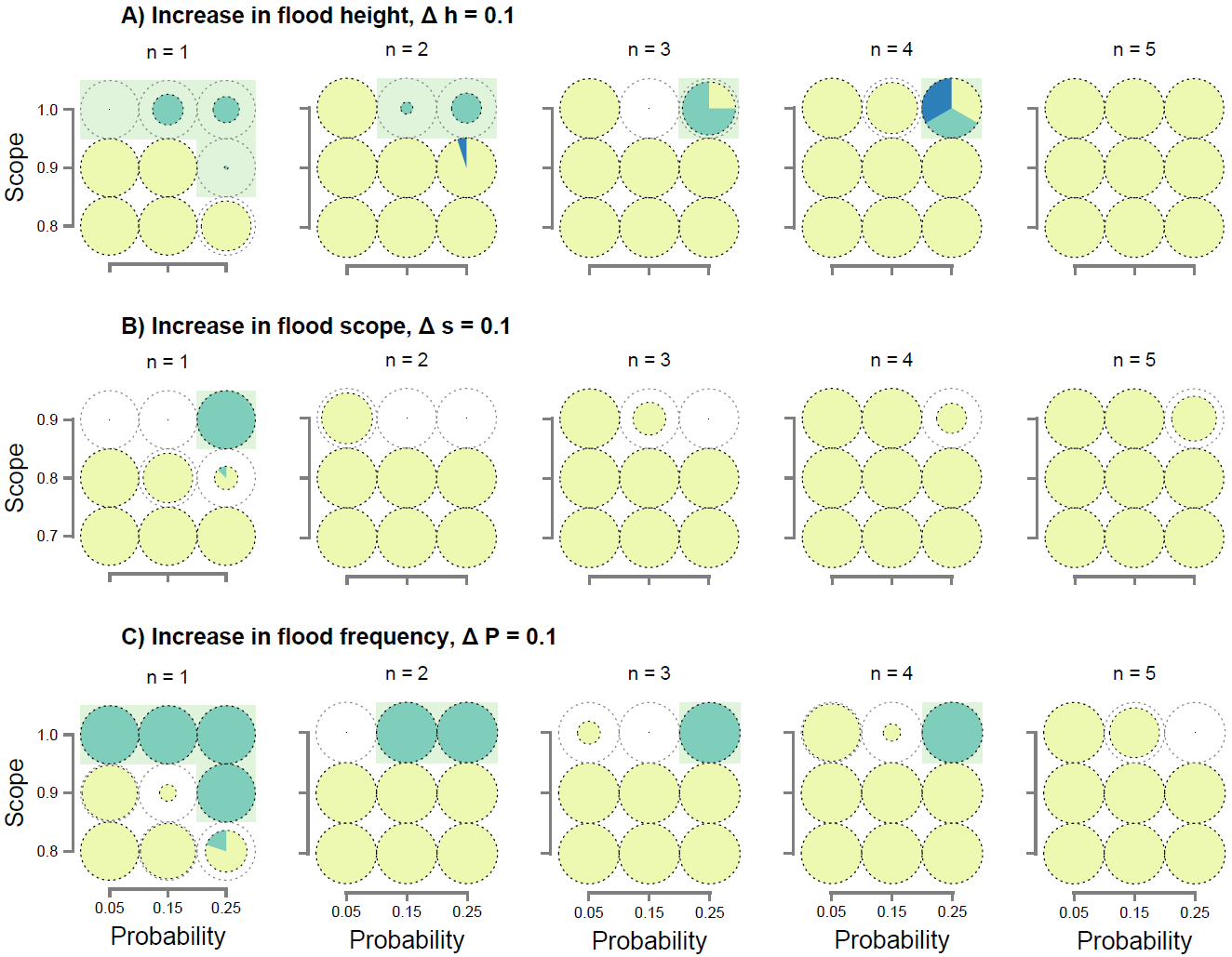


**Figure B.7.** Effect of climate change on population viability and phenotypic evolution when *c* = 2. In these two-stage simulations, 20 replicate populations are allowed to evolve for 2,000 generations under the combinations of flooding probability and scope depicted on the x- and y-axes and are subsequently subjected to a change in flooding regime. Fitness effects apply only to reproductive success. First row (A): Flood level is increased by 0.1. Second row (B): Scope of floods is increased by 0.1 (note different y-axis values). Third row (C): Frequency of floods is increased by 0.1. The diameter of each pie represents the proportion of populations that survived for 200 generations after the change in flooding regime out of the pool of populations that had survived up until the change. Colors within the pie charts represent mean nesting phenotypes of surviving populations (low-nesters in yellow, high-nesters in green, intermediate phenotypes in blue). For comparison, green shaded backgrounds indicate parameter combinations for which nesting above flood height evolved in more than 50 % of the corresponding simulations without climate change (i.e. locations in parameter space where pies were predominantly green in Figure B.6).


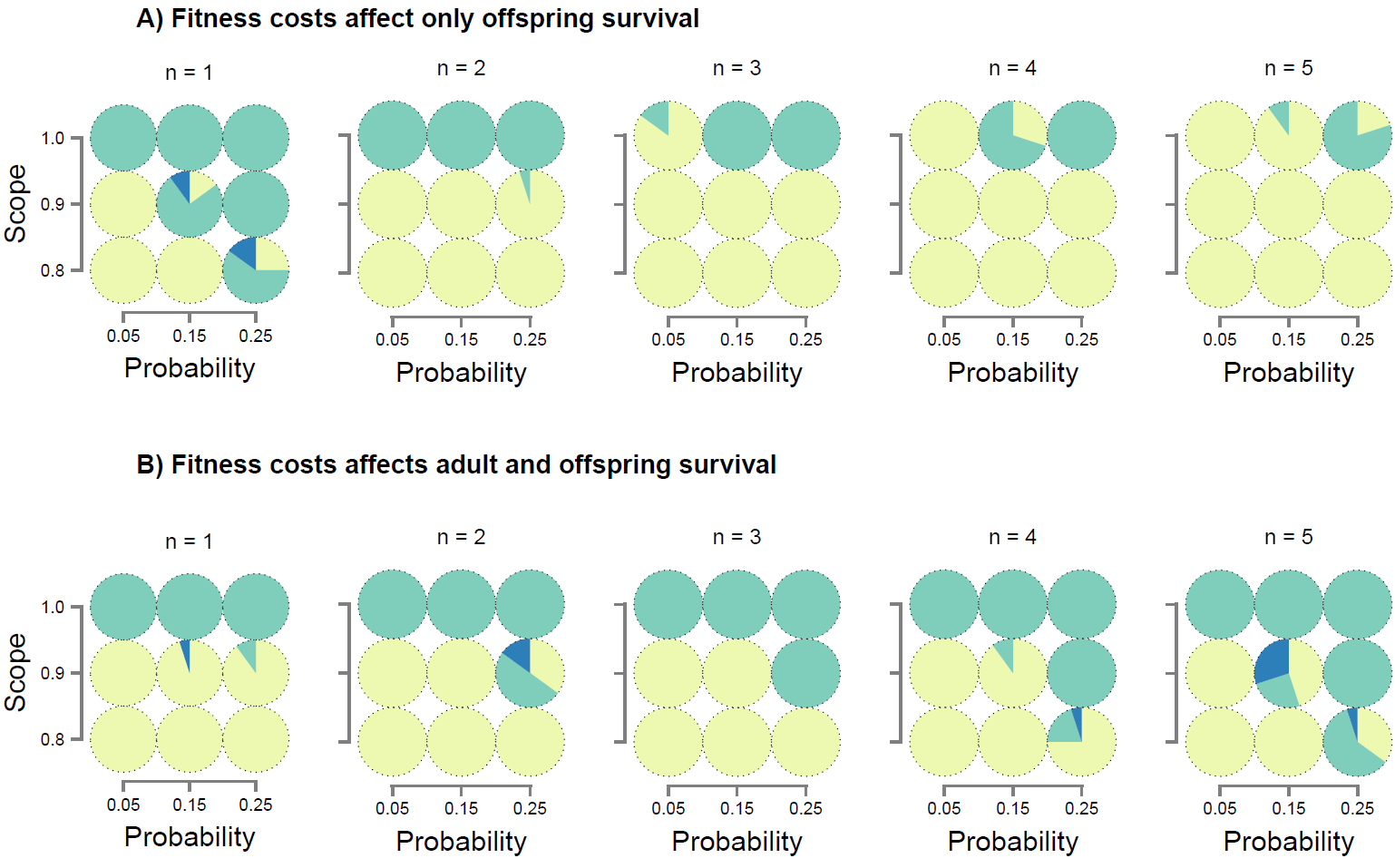


**Figure B.8.** Evolved nest heights after 2000 generations for populations with different numbers of reproductive events per lifetime, when mutations can switch phenotypes directly between high- and low-nesting. Rows indicate the target of fitness costs: In the top row (A), fitness costs affect only reproductive success, whereas in the bottom row (B) it affects adult survival as well as reproductive success. The top row is thus comparable with Figure 2 in the main text, and the bottom row with Figure B.1. The diameter of each pie depicts the proportion of 20 populations that survived until the end of the simulation (in this scenario, all populations survived at all parameter combinations). Colors indicate whether populations evolved low-nesting (yellow, $\bar{z}$< 0.05), high-nesting (green, $\bar{z}$ > *h*), or were polymorphic (blue, 0.05 ≤ $\bar{z}$ ≤ *h*).


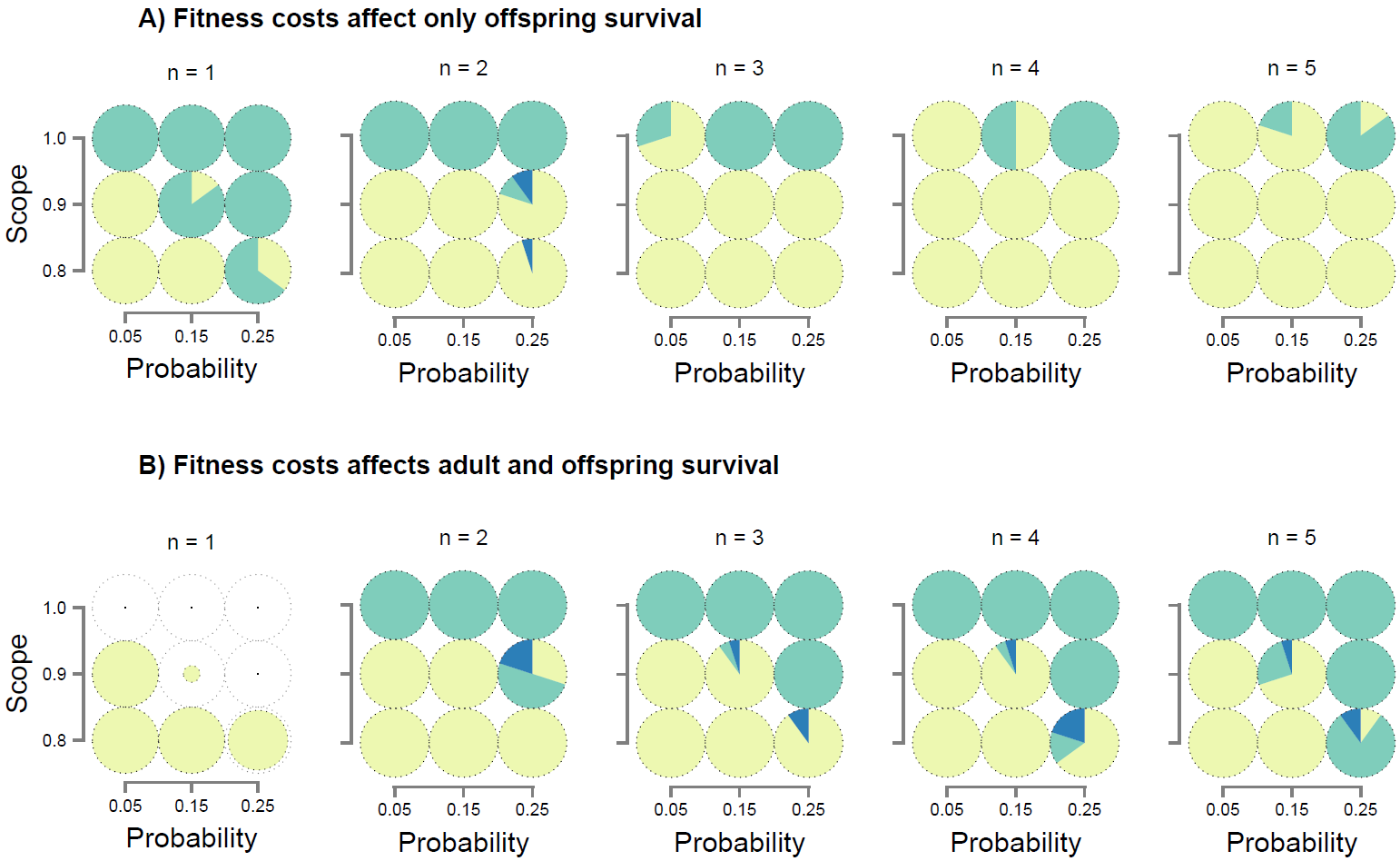


**Figure B.9.** Evolved nest heights after 2000 generations for populations with different numbers of reproductive events per lifetime, when mutations can switch phenotypes directly between high- and low-nesting, and clutch size *c* = 2. Rows indicate the target of fitness costs: In the top row (A), fitness costs affect only reproductive success, whereas in the bottom row (B) it affects adult survival as well as reproductive success. For the effect of changing *c*, the top row is thus comparable with Figure B.8a and the bottom row with Figure B.8b. For the effect of changing mutational effect, the top row is comparable with Figure 2 in the main text, and the bottom row with Figure B.1. The diameter of each pie depicts the proportion of 20 populations that survived until the end of the simulation. Colors indicate whether populations evolved low-nesting (yellow, $\bar{z}$< 0.05), high-nesting (green, $\bar{z}$ > *h*), or were polymorphic (blue, 0.05 ≤ $\bar{z}$ ≤ *h*).


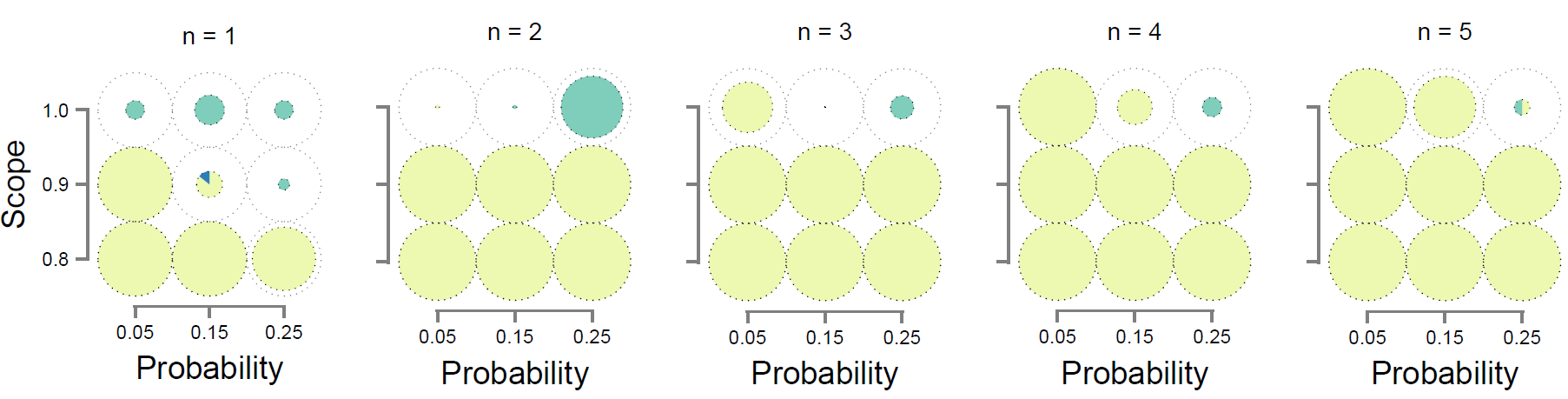


**Fig B.10.** Evolved nest heights after 2,000 generations when flood heights are variable. Other model assumptions are as in the baseline version of the model (so the pies are directly comparable with Figure 2 in the main text): Generations are discrete, fitness effects of flooding and predation apply to only nestling survival, and mutations have small effects.

Appendix C: Evolutionary trajectories

To illustrate the evolutionary trajectories of mean nest height over time, we produced plots depicting each replicate population trajectory in an opaque red line, such that darker red areas represent evolutionary trajectories taken by multiple independent populations at a given time. Figures C.1-5 show some examples that illustrate key dynamics occurring at particular parameter combinations and climate change scenarios, as well as some examples comparing the evolutionary trajectories created of the models with discrete versus overlapping generations. In the figure legends we briefly mention the relevant processes creating the depicted dynamics. We also plot transparent blue lines representing within-population genetic variance (altered y-axis scale on right). Note that a polymorphic population consisting of equal parts low- and high-nesting individuals will have a genetic variance of 0.04, i.e. the blue lines are on the same y-axis height of the red lines of high-nesting phenotypes. Solid black lines depict the mean of all live populations as a point of comparison. Dashed vertical lines are used in scenarios with climate change to indicate the time at which a change in flooding severity, scope or frequency is introduced (i.e., generation 2000).

Information about supplementary datasets S1 and S2: We also provide in Supplementary Materials the datasets S1 and S2 complete sets of evolutionary trajectory plots for all parameter combinations in the discrete generations model, which are uploaded to Dryad. As for Figures C.1-5 here, these plots were created by depicting the trajectories of every replicate simulation in slightly transparent red lines. Thus, darker red sections in these plots indicate trajectories that were shared by more independent simulations at a given time. Transparent blue lines represent within-generation population variance (altered y-axis on right).

The supplementary datasets are titled “Evolutionary trajectories Only nestling mortality” and “Evolutionary trajectories Both fitness effects” (the latter containing simulations in which fitness effects apply to both offspring and adult mortality). The header for each page indicates the type of climate change effect (“None”, “Height of floods”, “Scope of floods” or “Probability of floods”) and the number of broods per year (1-5). The 9 subpanels on each page are organized in the same way as the pie plots shown in the main text, with the rows corresponding to different scopes of floods, and columns corresponding to different probabilities of floods. Parameter values are stated above each subpanel. Note that in the case of climate change effects, values refer to pre-climate change conditions.


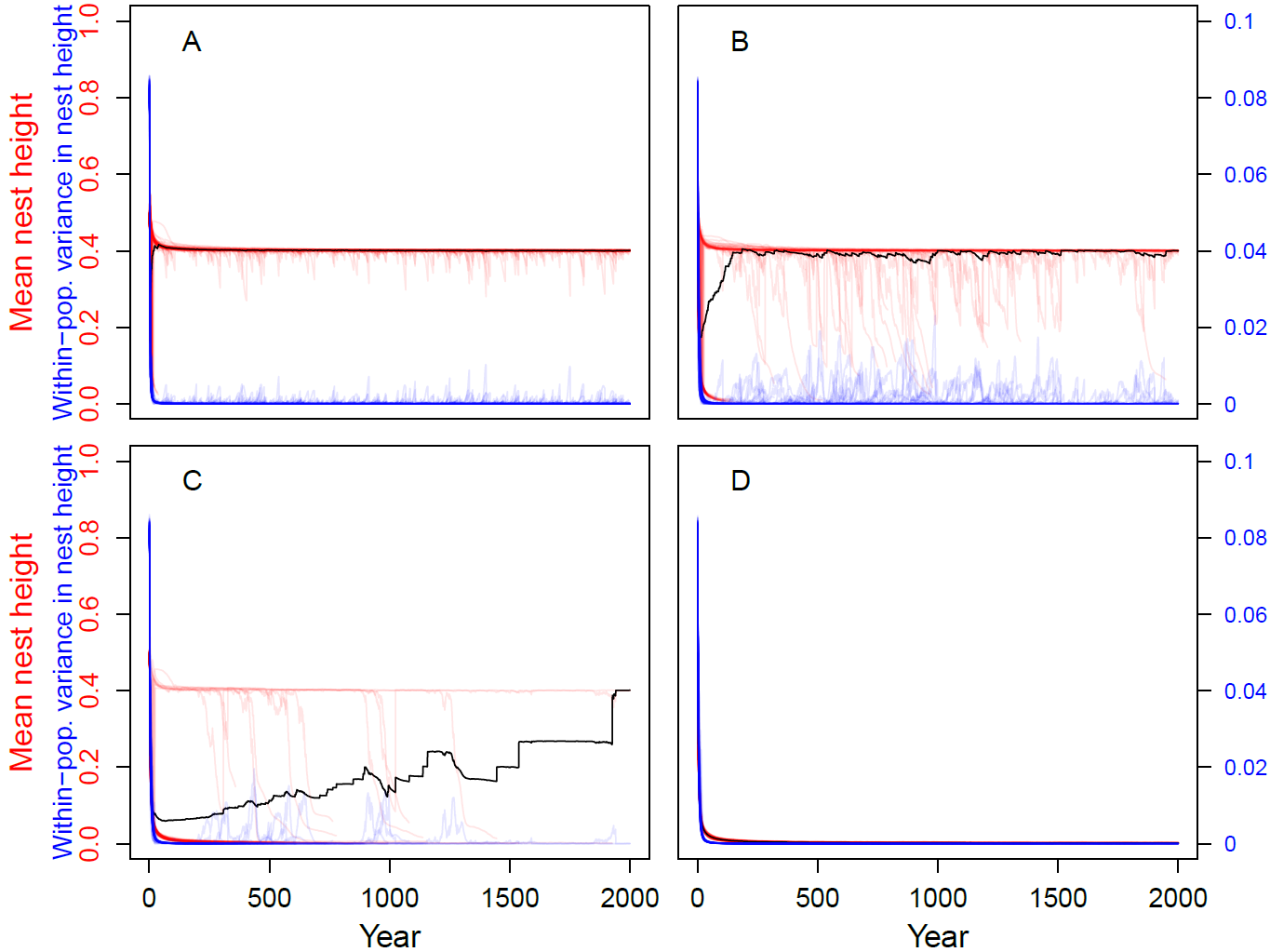


**Figure C.1** Evolutionary trajectories of mean nest height (opaque red lines) and variance in nest height (opaque blue lines) over time in 100 replicate populations, and average trajectories across all live populations (solid black line). In this scenario, probability of floods P = 0.15, scope of floods s = 1, and other baseline model assumptions apply. Populations produce (A) 1, (B) 2, (C) 3 and (D) 4 broods per year. In (A), nest height stabilizes above the flood height within the first 100 generations. There are only intermittent decreases in nest height, driven by the competitive advantage (less nest predation) of nesting lower, in the interim between floods, but once the next flood occurs the population returns to nesting above the flood height. In (B), nest height initially exhibits two distinct patterns, low-nesting or high-nesting, but the low-nesters always go extinct within a few hundred generations. Producing more broods per year allows a stronger advantage to low-nesting, since even if a flood does occur and kill one brood, the other may still survive, and the genes for low-nesting phenotypes can persist in the population. However, sooner or later the ‘perfect flood’ occurs affecting both nests in a year, and only high-nesting lineages are able to survive these. The red lines descending from the flood height represent populations that end up going extinct. In (C), the chance of a ‘perfect flood’ affecting all three broods in a year is much lower, so low-nesters are able to persist for much longer. For the first 1000 generations low-nesting populations are in the majority, but as they gradually go extinct, only high-nesting populations remain at the end of the simulation. In (D), however, high-nesting does not evolve, since the advantage to low-nesting becomes even larger.


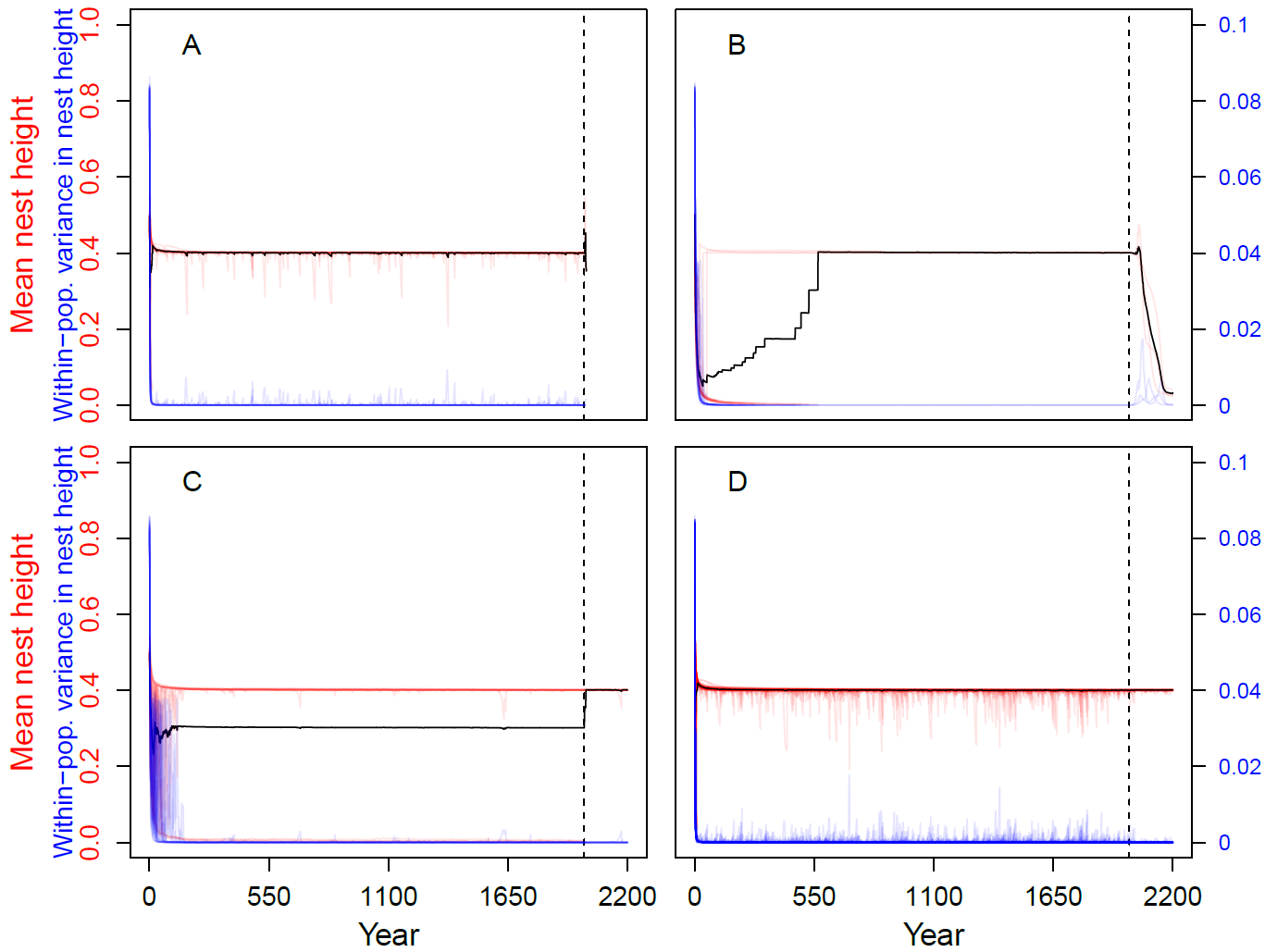
**Figure C.2.** Effects of climate change on evolutionary trajectories of mean nest height (opaque red lines) and variance in nest height (opaque blue lines) over time in 100 replicate populations, and average trajectory across all live populations (solid black line). Baseline model assumptions apply, and individuals produce one brood per year, except in panel B. Up until year 2000 (dashed vertical line), parameter settings are (A) probability of floods *P* = 0.25, scope of floods *s* = 1, fitness costs affect both adult and offspring survival; (B) *P* = 0.25, *s* = 1, populations produce four broods per year, fitness costs affect only offspring; (C) *P* = 0.25, *s* = 0.9, fitness costs affect only offspring; (D) *P* = 0.15, *s* = 1, fitness costs affect both adult and offspring survival. After 2,000 years climate change is introduced, causing a shift in the flooding regimes: (A) Flood level increases from 0.4 to 0.5, causing all populations to go extinct. (B) As in A, but producing more broods per year allows some lineages to evolve lower nest heights, since the floods very rarely affect all four broods in a year, but as the trajectories from the beginning of the simulations indicate, it is only a matter of time before these lineages will go extinct as well. (C) Scope of floods increases from 0.9 to 1, causing all low-nesters to go extinct immediately, but leaving high-nesters unaffected. (D) Probability of floods *P* increases from 0.15 to 0.35, which has the effect of stabilizing the populations to nest above the flood level, whereas rarer floods caused populations to drift to lower flood heights (and subsequently go extinct when the next flood occurred).


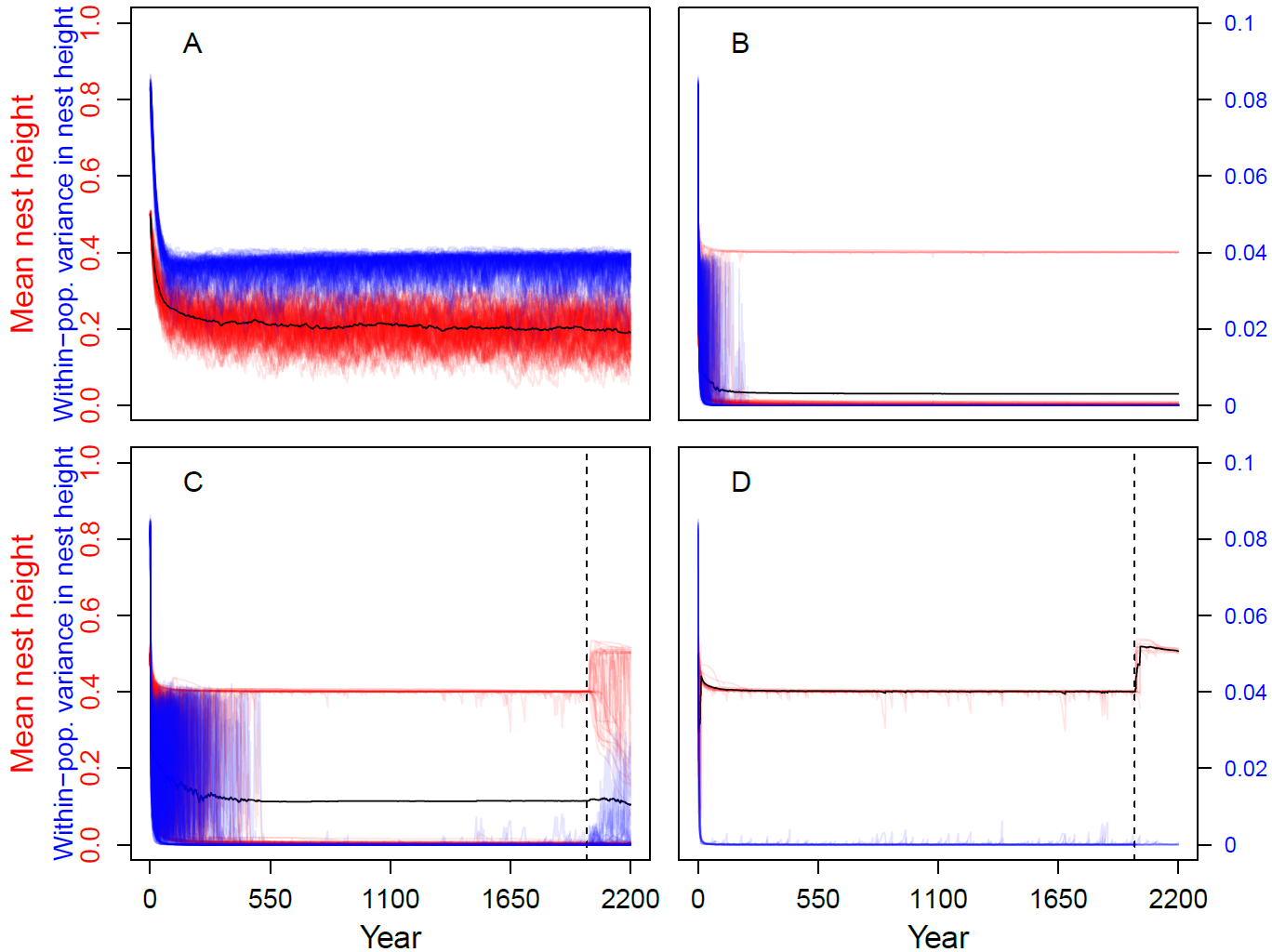
**Figure C.3.** Evolutionary trajectories of mean nest height (opaque red lines) and variance in nest height (opaque blue lines) over time in 100 replicate populations, and average trajectory across all live populations (solid black line). The top row compares the effects of target of fitness costs in the overlapping generations model. Results shown are for *α* = 0.1, *s* = 0.9 and *P* = 0.25. In (A), fitness costs only affect offspring survival, whereas in (B) fitness costs affect adult survival as well as offspring survival. The high within-population variance in (A) indicate that a genetic polymorphism is maintained, but this does not happen in (B). The bottom row compares the effect of discrete versus overlapping generations when climate change after 2,000 years (dashed vertical line) increases flood height from 0.4 to 0.5. *P* = 0.85, *s* = 1, and fitness affects only offspring survival. (C): Overlapping generations, *α* = 0.8, populations produce one brood per year. (D): Discrete generations, populations produce 2 broods per year. Despite having fewer expected breeding events within a lifetime (1/*α* = 1.25), the populations with overlapping generations are more often able to survive the change in flood height, since the lineage does not go extinct despite an entire year with no recruitment. Also, notice that genetic polymorphisms are not maintained in C despite overlapping generations and fitness costs only affecting offspring – a consequence of shorter adult lifespans than in A.


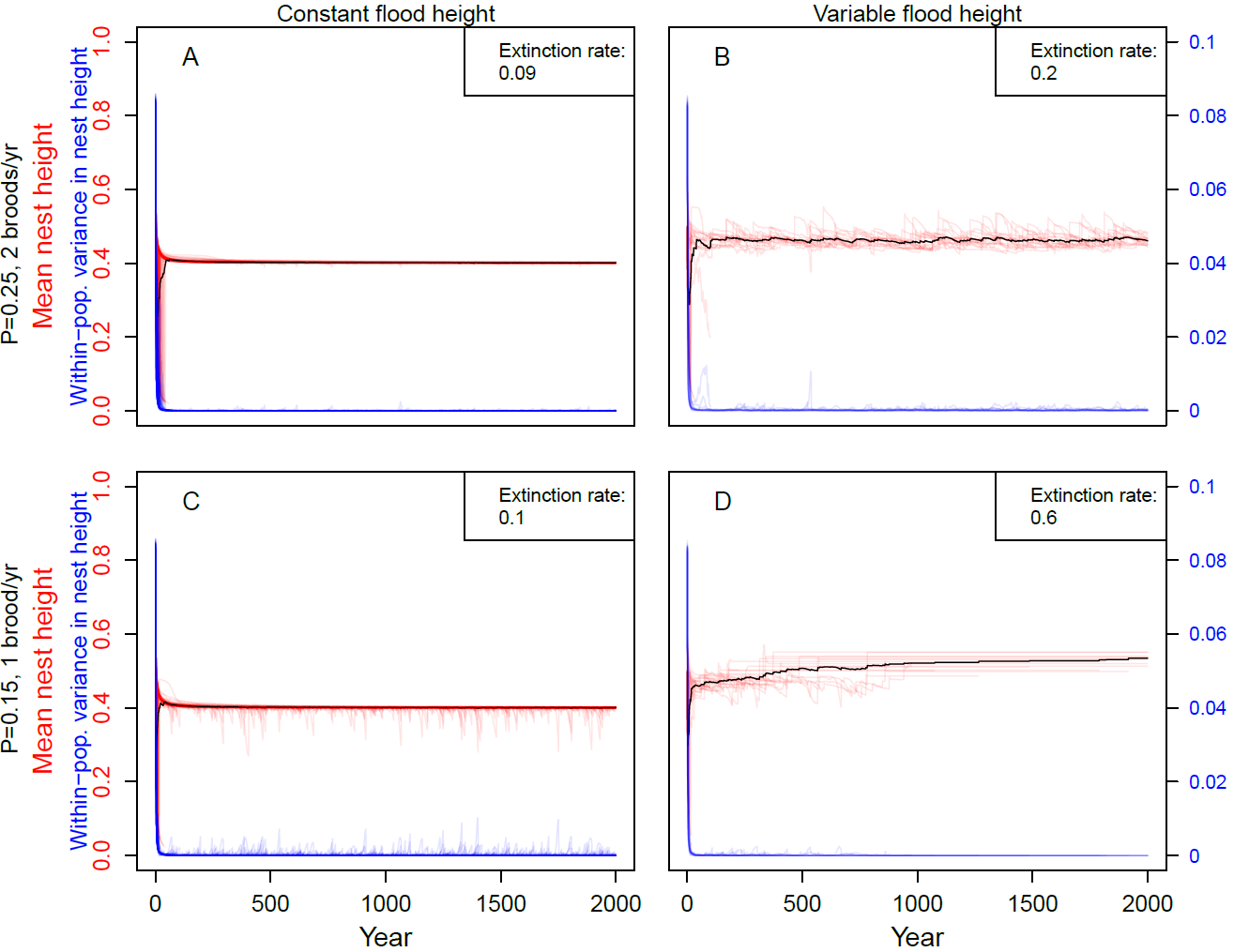


**Figure C.4.** Comparing the effect of constant versus variable flood heights by showing evolutionary trajectories of mean nest height (opaque red lines) and variance in nest height (opaque blue lines) over time in replicate populations. Average trajectories across all live populations (solid black line). First row (A, C): Height of floods (when they occur) are kept constant, *h* = 0.4. Second row (B, D): Height of floods is variable with mean *h* = 0.4 and standard deviation *h*/10 = 0.04. In all panels, fitness effects apply to only nestling survival, generations are discrete, and flooding scope *s* = 1. Top row (A, B): Probability of floods *P* = 0.25, individuals can have two broods per year. Bottom row (C, D): *P* = 0.15, individuals have one brood per year. Panel A thus corresponds to Figure 2, top right pie in panel *n* = 2, and panel B to Figure 2, top middle pie in panel *n* = 1.


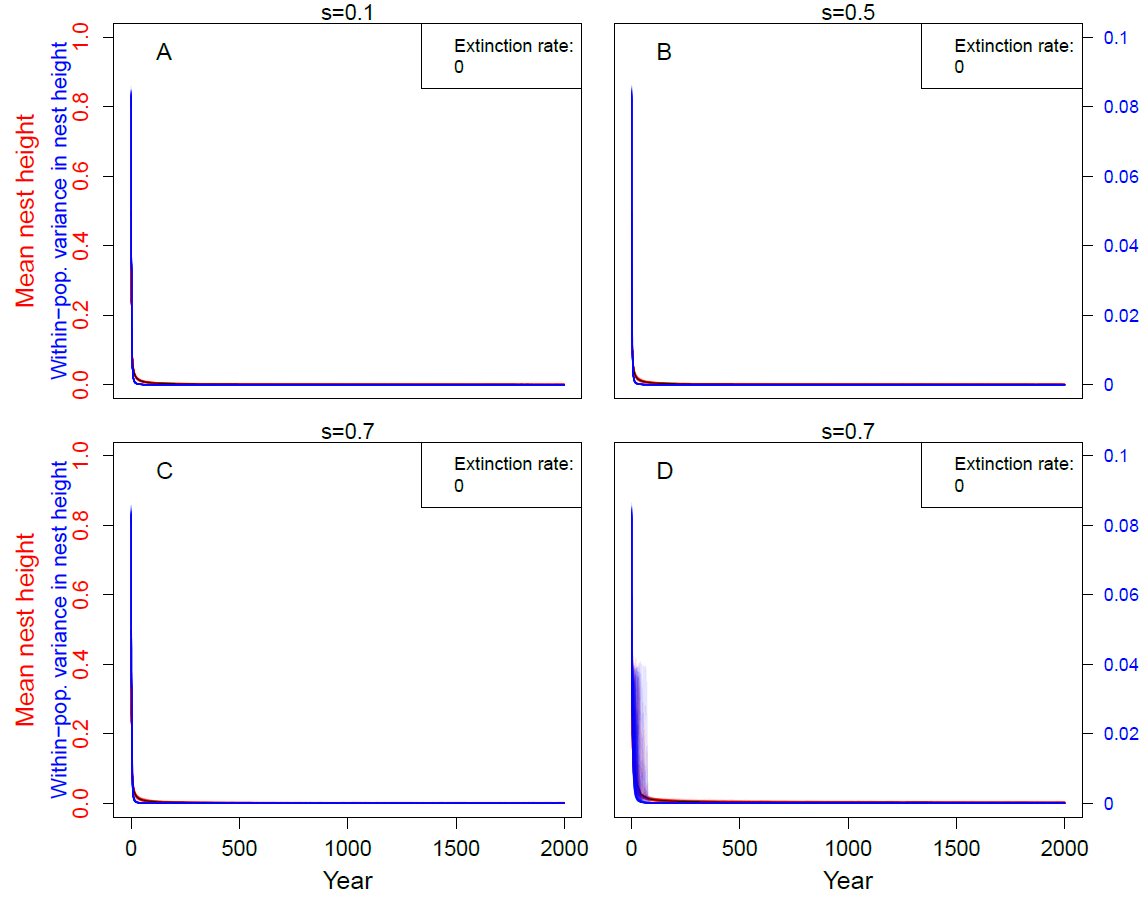


**Figure C.5.** Evolutionary trajectories of mean nest height (opaque red lines) and variance in nest height (opaque blue lines) over time in 100 replicate populations, and average trajectory across all live populations (solid black line) when floods affect a smaller proportion (*s*) of the population. (A): *s* = 0.1, (B): *s* = 0.5, (C) and (D): *s* = 0.7. Baseline model assumptions apply, except in (D) where flooding affects both offspring and adult survival. In all plots, individuals have one brood per lifetime, and flood frequency is 0.25 (i.e., scenarios strongly favoring high-nesting for larger flooding scopes).
