# Supplementary material for: Alternative responses to rare selection events are differentially vulnerable to changes in the frequency, scope, and intensity of environmental extremes
Source: Ecol Evol. 2019 Sep 27;9(20):11752–61. doi: 10.1002/ece3.5675 (PMC6822052; doi:10.1002/ece3.5675)
Supplement: Supplementary file 2 [file ECE3-9-11752-s002.pdf]

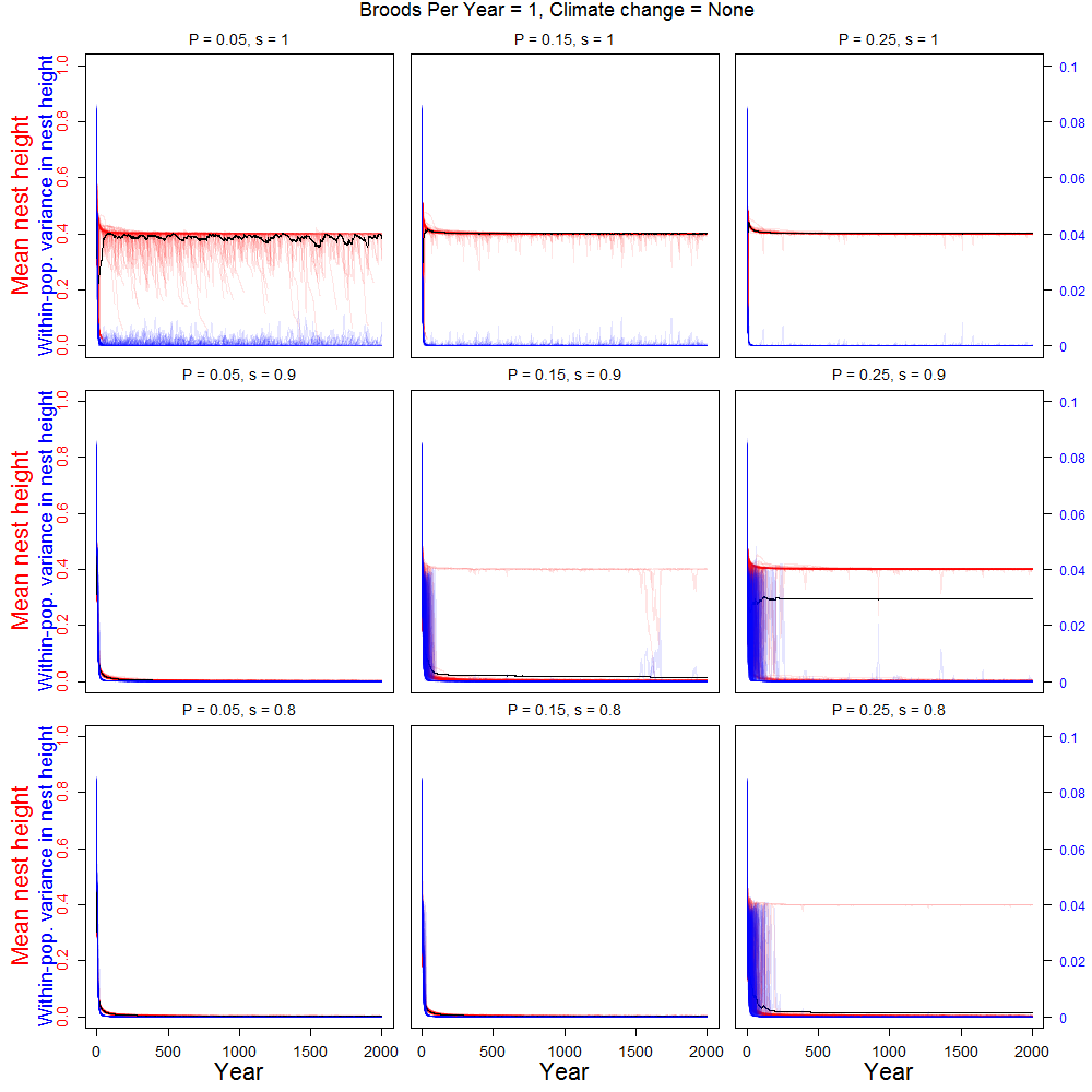

Broods Per Year = 1, Climate change = None
P = 0.05, s = 1
P = 0.15, s = 1
P = 0.25, s = 1
P = 0.05, s = 0.9
P = 0.15, s = 0.9
P = 0.25, s = 0.9
P = 0.05, s = 0.8
P = 0.15, s = 0.8
P = 0.25, s = 0.8
Mean nest height
Within-pop. variance in nest height
0.0
0.2
0.4
0.6
0.8
1.0
0
0.02
0.04
0.06
0.08
0.1
0
500
1000
1500
2000
Year

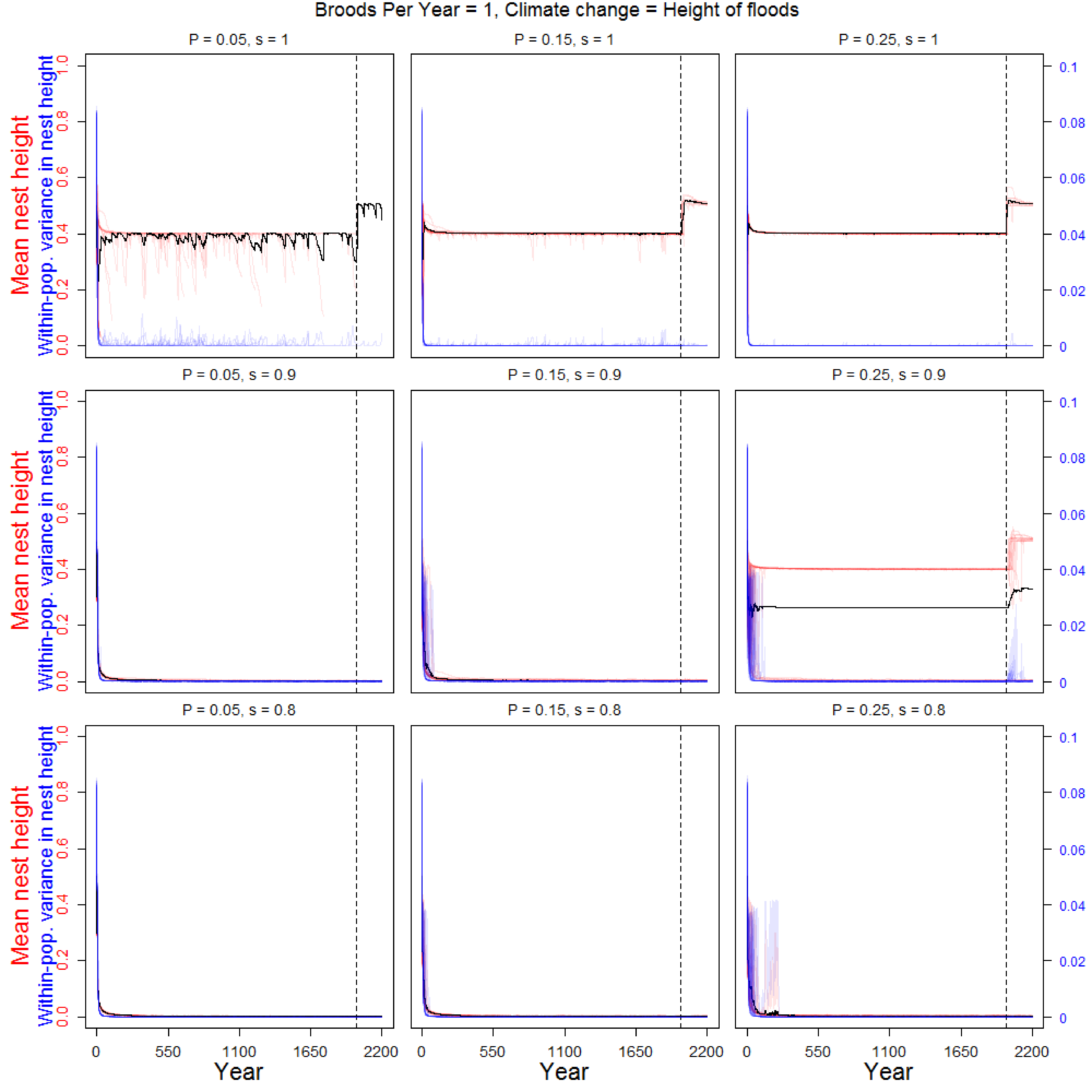

Broods Per Year = 1, Climate change = Height of floods
P = 0.05, s = 1
P = 0.15, s = 1
P = 0.25, s = 1
P = 0.05, s = 0.9
P = 0.15, s = 0.9
P = 0.25, s = 0.9
P = 0.05, s = 0.8
P = 0.15, s = 0.8
P = 0.25, s = 0.8
Mean nest height
Within-pop. variance in nest height
0.0
0.2
0.4
0.6
0.8
1.0
0
0.02
0.04
0.06
0.08
0.1
0
550
1100
1650
2200
Year

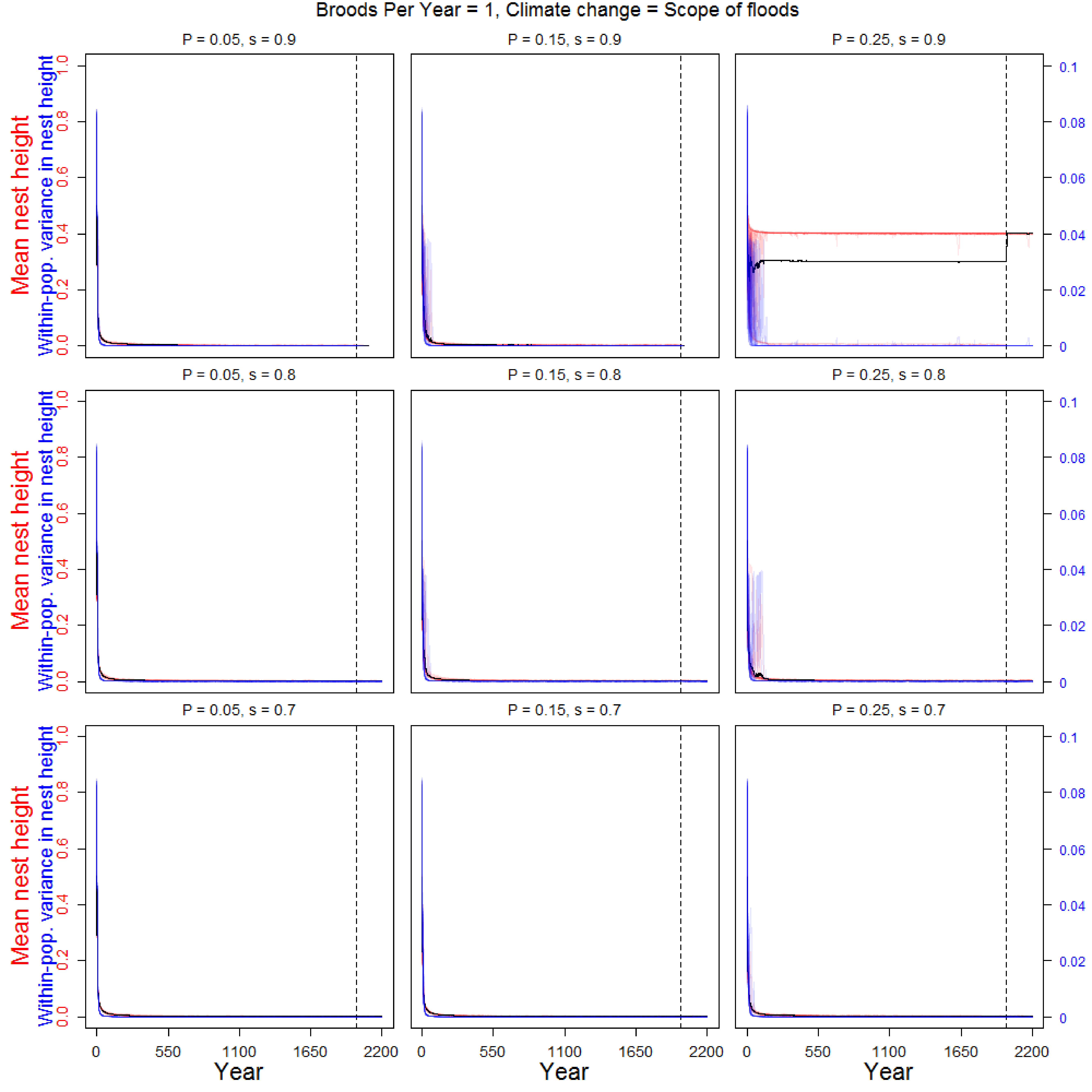

Broods Per Year = 1, Climate change = Scope of floods
P = 0.05, s = 0.9
P = 0.15, s = 0.9
P = 0.25, s = 0.9
P = 0.05, s = 0.8
P = 0.15, s = 0.8
P = 0.25, s = 0.8
P = 0.05, s = 0.7
P = 0.15, s = 0.7
P = 0.25, s = 0.7
Mean nest height
Within-pop. variance in nest height
0.0
0.2
0.4
0.6
0.8
1.0
0
0.02
0.04
0.06
0.08
0.1
0
550
1100
1650
2200
Year

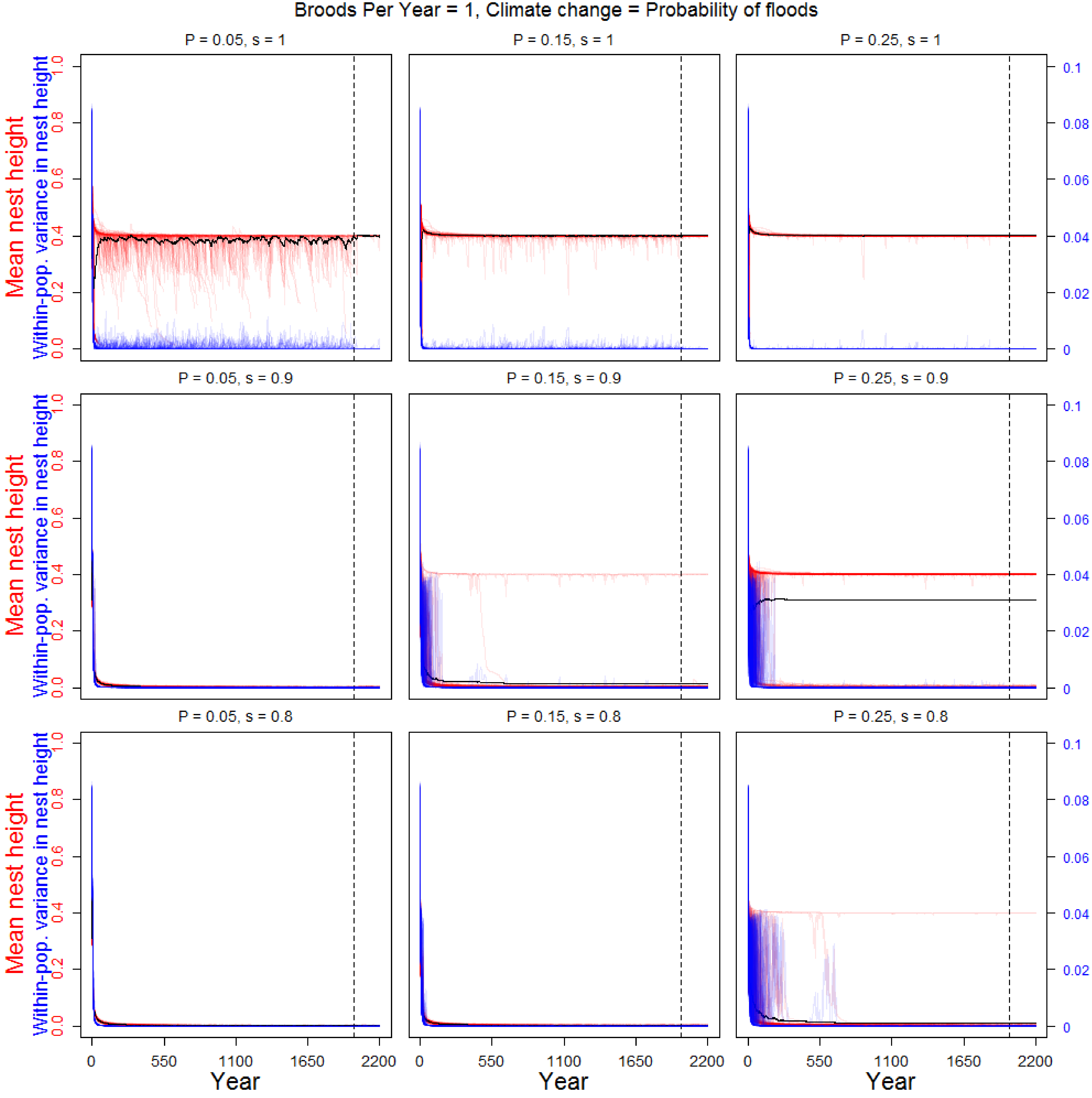
Broods Per Year = 1, Climate change = Probability of floods
P = 0.05, s = 1
P = 0.15, s = 1
P = 0.25, s = 1
P = 0.05, s = 0.9
P = 0.15, s = 0.9
P = 0.25, s = 0.9
P = 0.05, s = 0.8
P = 0.15, s = 0.8
P = 0.25, s = 0.8
Mean nest height
Within-pop. variance in nest height
Year
0
550
1100
1650
2200
0.0
0.2
0.4
0.6
0.8
1.0
0
0.02
0.04
0.06
0.08
0.1

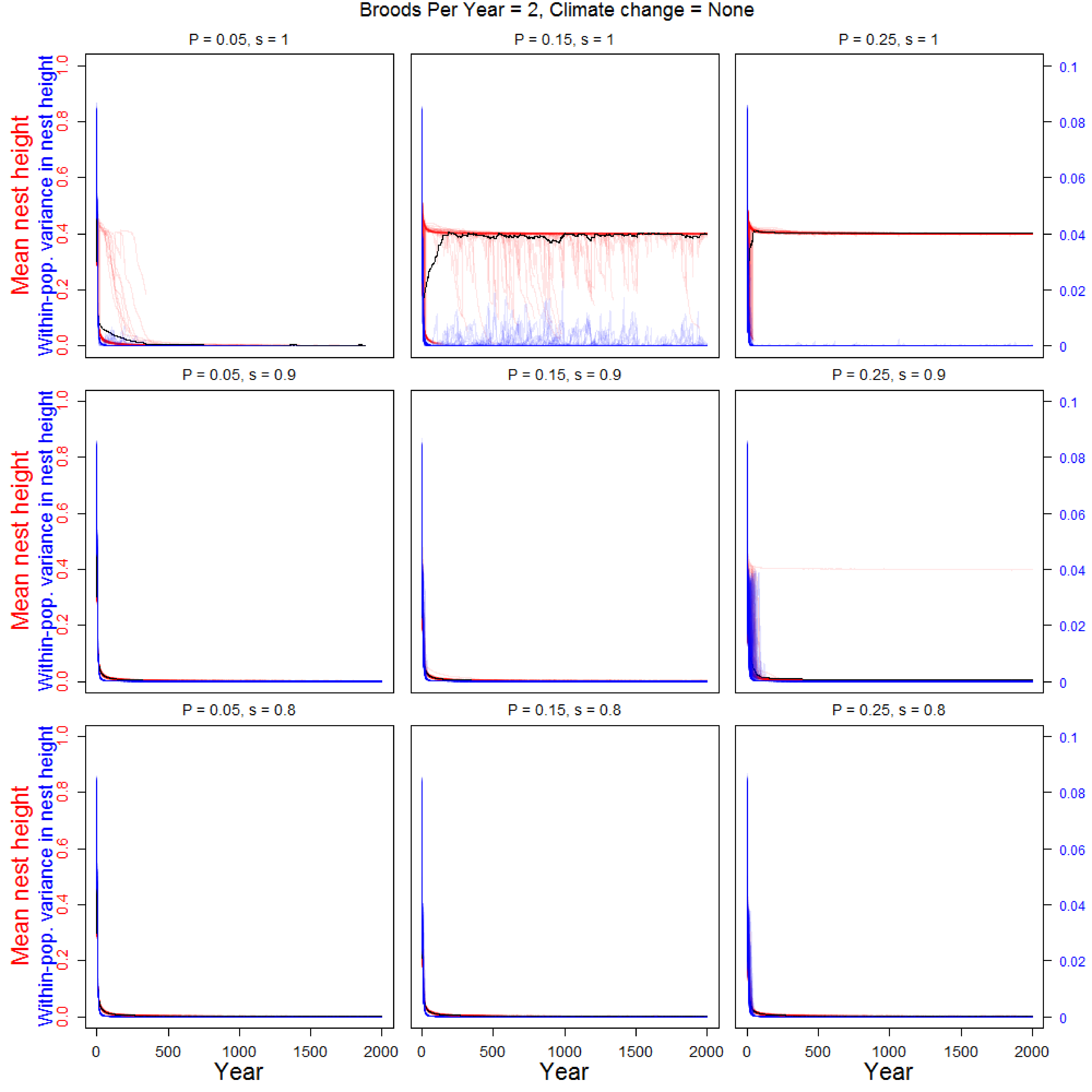

Broods Per Year = 2, Climate change = None
P = 0.05, s = 1
P = 0.15, s = 1
P = 0.25, s = 1
P = 0.05, s = 0.9
P = 0.15, s = 0.9
P = 0.25, s = 0.9
P = 0.05, s = 0.8
P = 0.15, s = 0.8
P = 0.25, s = 0.8
Mean nest height
Within-pop. variance in nest height
0.0
0.2
0.4
0.6
0.8
1.0
0
0.02
0.04
0.06
0.08
0.1
0
500
1000
1500
2000
Year

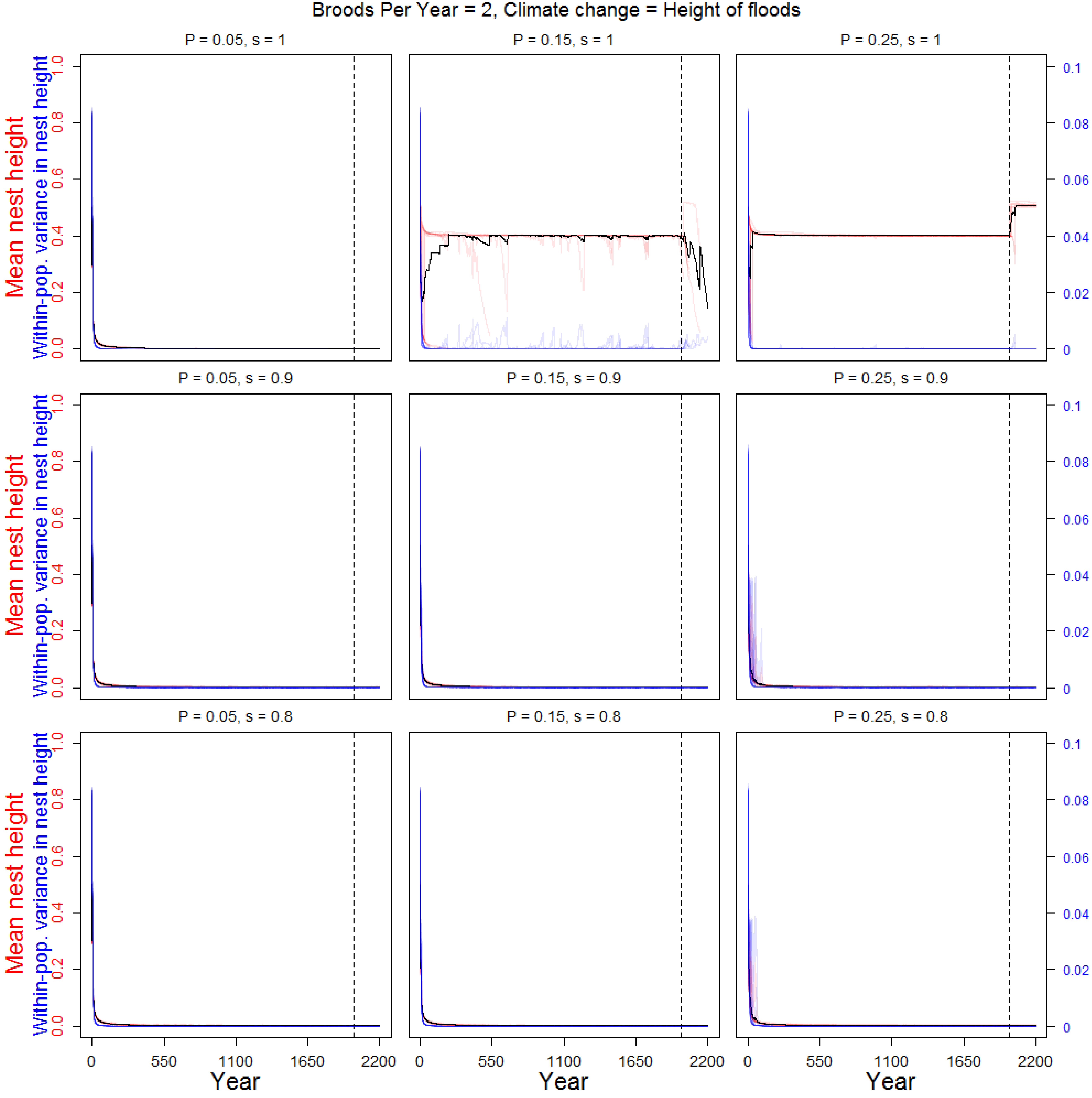

Broods Per Year = 2, Climate change = Height of floods
P = 0.05, s = 1
P = 0.15, s = 1
P = 0.25, s = 1
P = 0.05, s = 0.9
P = 0.15, s = 0.9
P = 0.25, s = 0.9
P = 0.05, s = 0.8
P = 0.15, s = 0.8
P = 0.25, s = 0.8
Mean nest height
Within-pop. variance in nest height
0.0
0.2
0.4
0.6
0.8
1.0
0
0.02
0.04
0.06
0.08
0.1
0
550
1100
1650
2200
Year

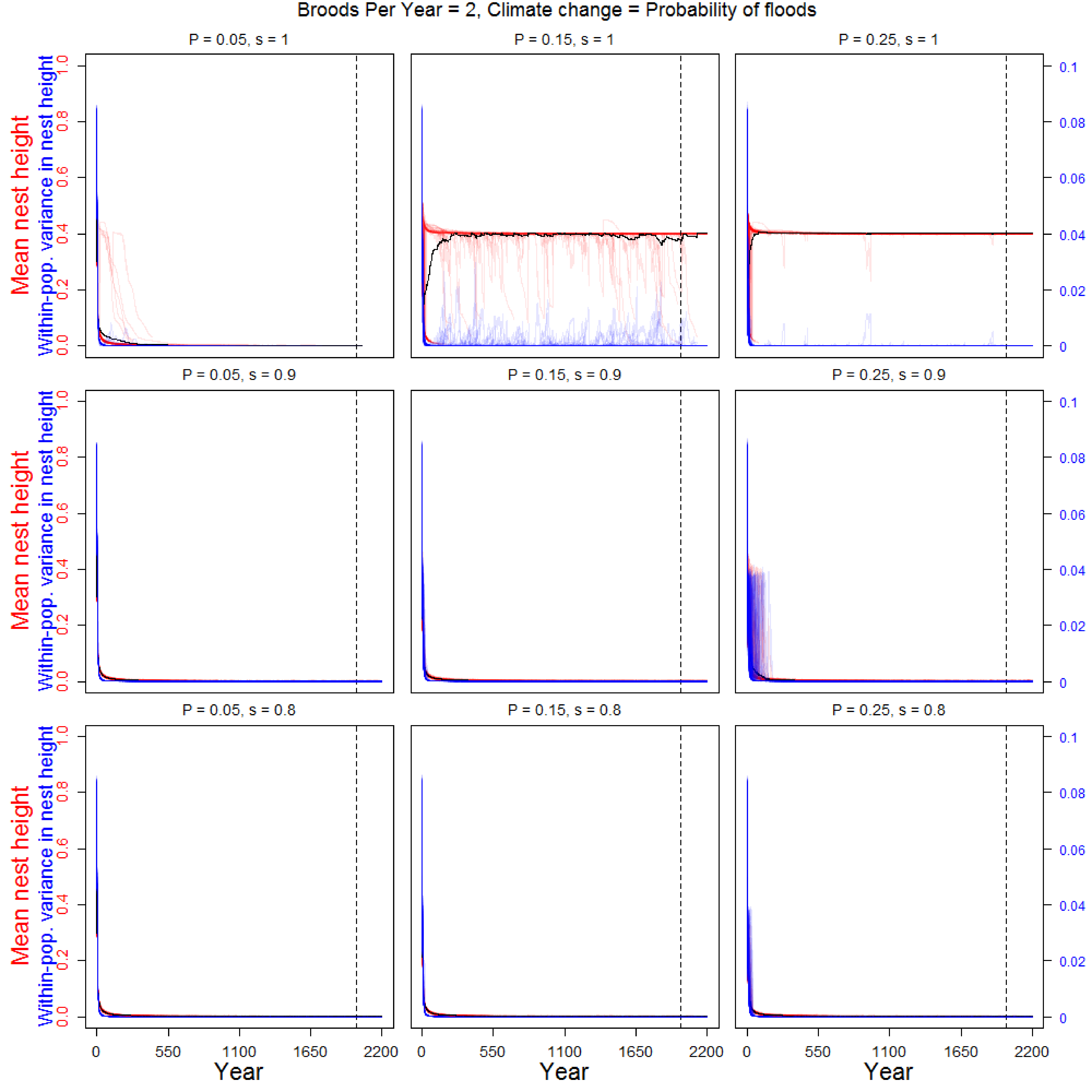

Broods Per Year = 2, Climate change = Probability of floods
P = 0.05, s = 1
P = 0.15, s = 1
P = 0.25, s = 1
P = 0.05, s = 0.9
P = 0.15, s = 0.9
P = 0.25, s = 0.9
P = 0.05, s = 0.8
P = 0.15, s = 0.8
P = 0.25, s = 0.8
Mean nest height
Within-pop. variance in nest height
0.0
0.2
0.4
0.6
0.8
1.0
0
0.02
0.04
0.06
0.08
0.1
0
550
1100
1650
2200
Year

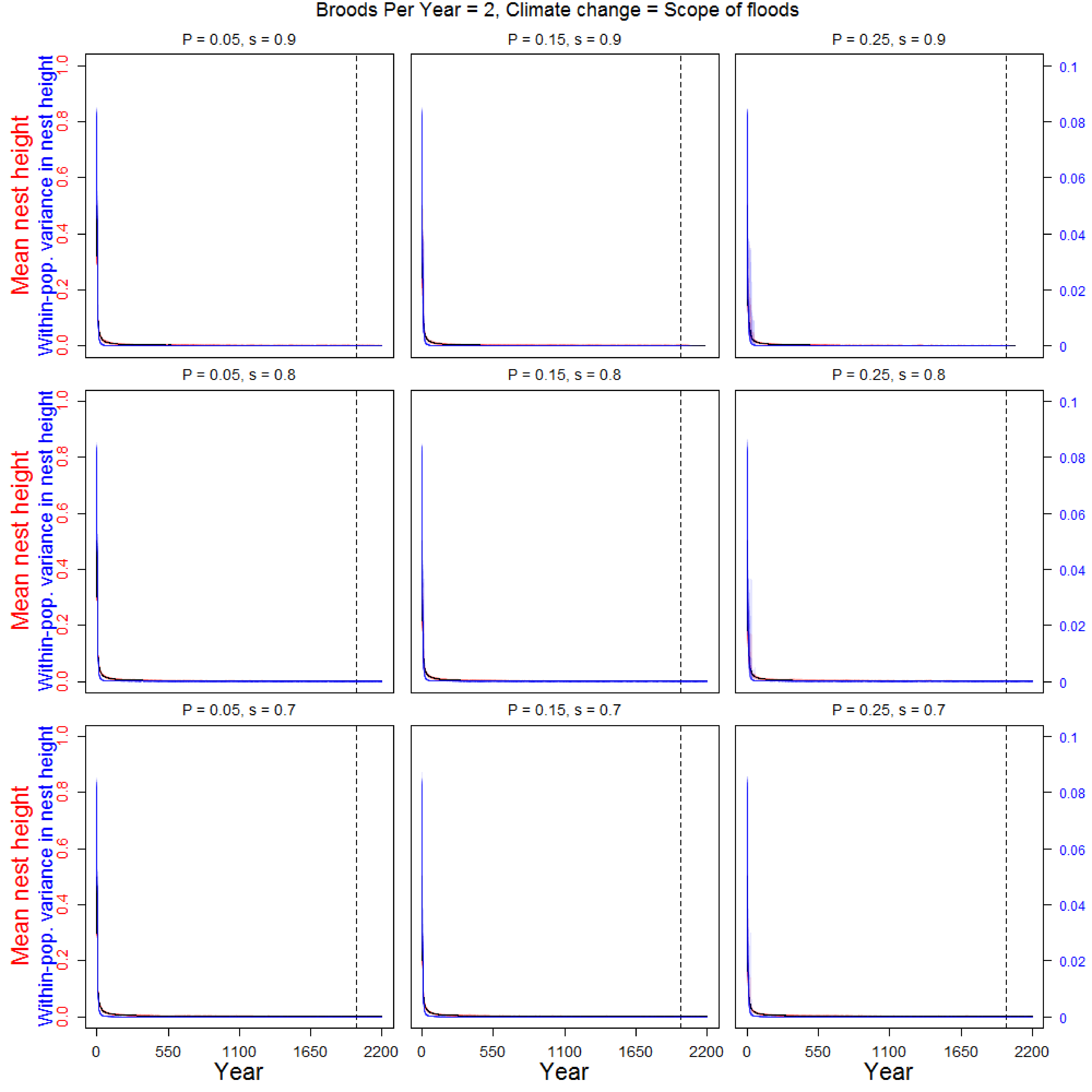
Broods Per Year = 2, Climate change = Scope of floods
P = 0.05, s = 0.9
P = 0.15, s = 0.9
P = 0.25, s = 0.9
P = 0.05, s = 0.8
P = 0.15, s = 0.8
P = 0.25, s = 0.8
P = 0.05, s = 0.7
P = 0.15, s = 0.7
P = 0.25, s = 0.7
Mean nest height
Within-pop. variance in nest height
0.0
0.2
0.4
0.6
0.8
1.0
0
0.02
0.04
0.06
0.08
0.1
0
550
1100
1650
2200
Year

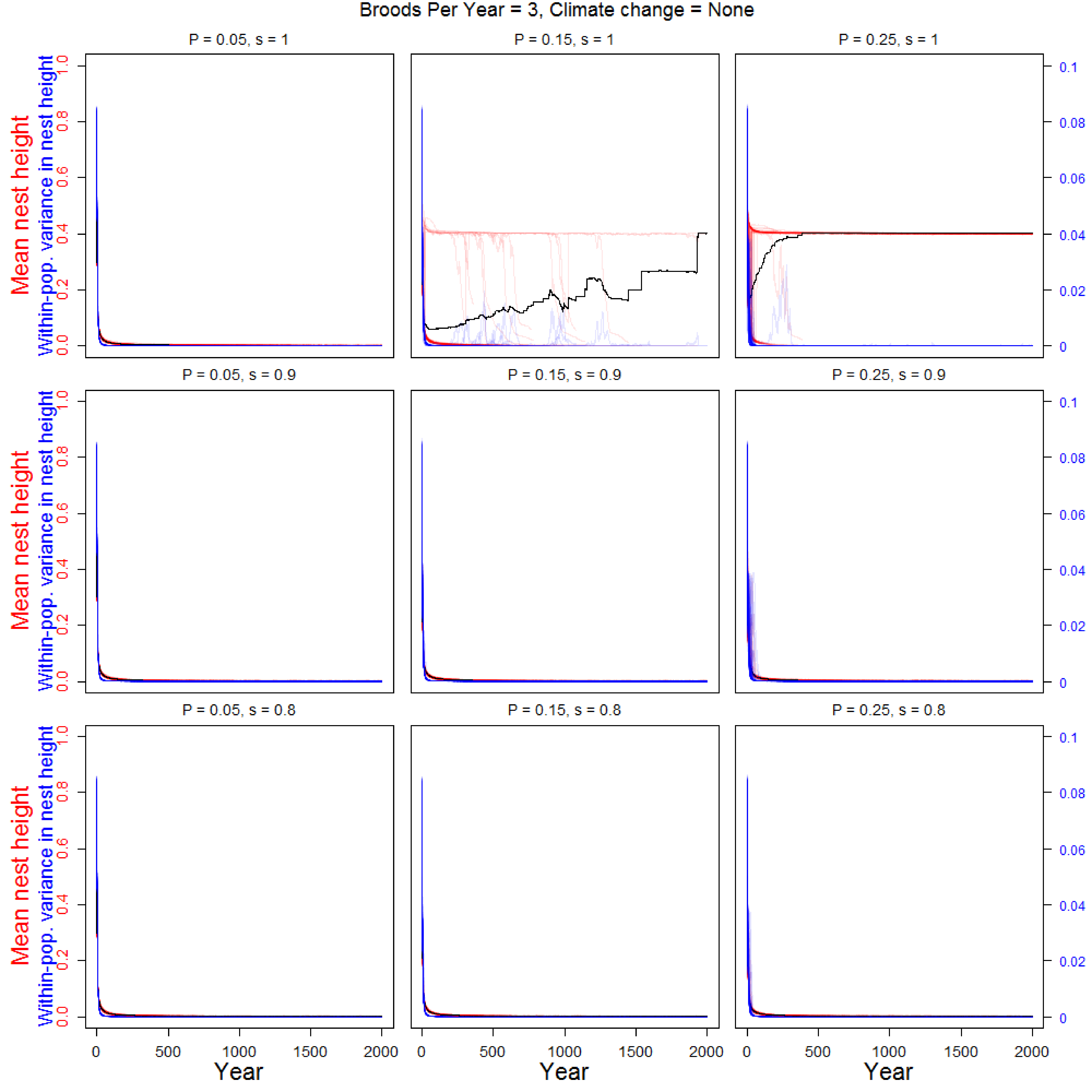

Broods Per Year = 3, Climate change = None
P = 0.05, s = 1
P = 0.15, s = 1
P = 0.25, s = 1
P = 0.05, s = 0.9
P = 0.15, s = 0.9
P = 0.25, s = 0.9
P = 0.05, s = 0.8
P = 0.15, s = 0.8
P = 0.25, s = 0.8
Mean nest height
Within-pop. variance in nest height
0.0
0.2
0.4
0.6
0.8
1.0
0
0.02
0.04
0.06
0.08
0.1
0
500
1000
1500
2000
Year

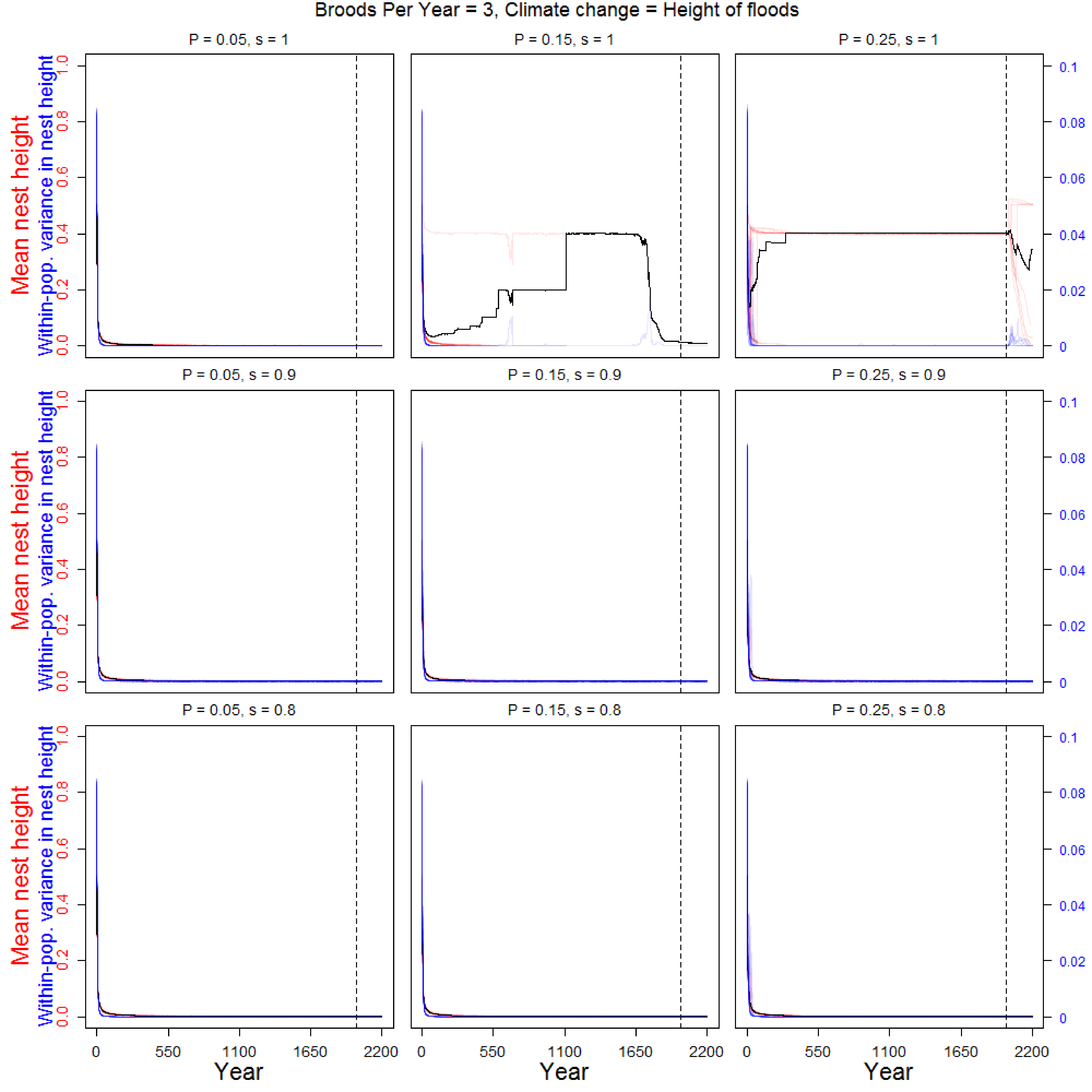

Broods Per Year = 3, Climate change = Height of floods
P = 0.05, s = 1
P = 0.15, s = 1
P = 0.25, s = 1
P = 0.05, s = 0.9
P = 0.15, s = 0.9
P = 0.25, s = 0.9
P = 0.05, s = 0.8
P = 0.15, s = 0.8
P = 0.25, s = 0.8
Mean nest height
Within-pop. variance in nest height
Year
0
550
1100
1650
2200
0.0
0.2
0.4
0.6
0.8
1.0
0
0.02
0.04
0.06
0.08
0.1

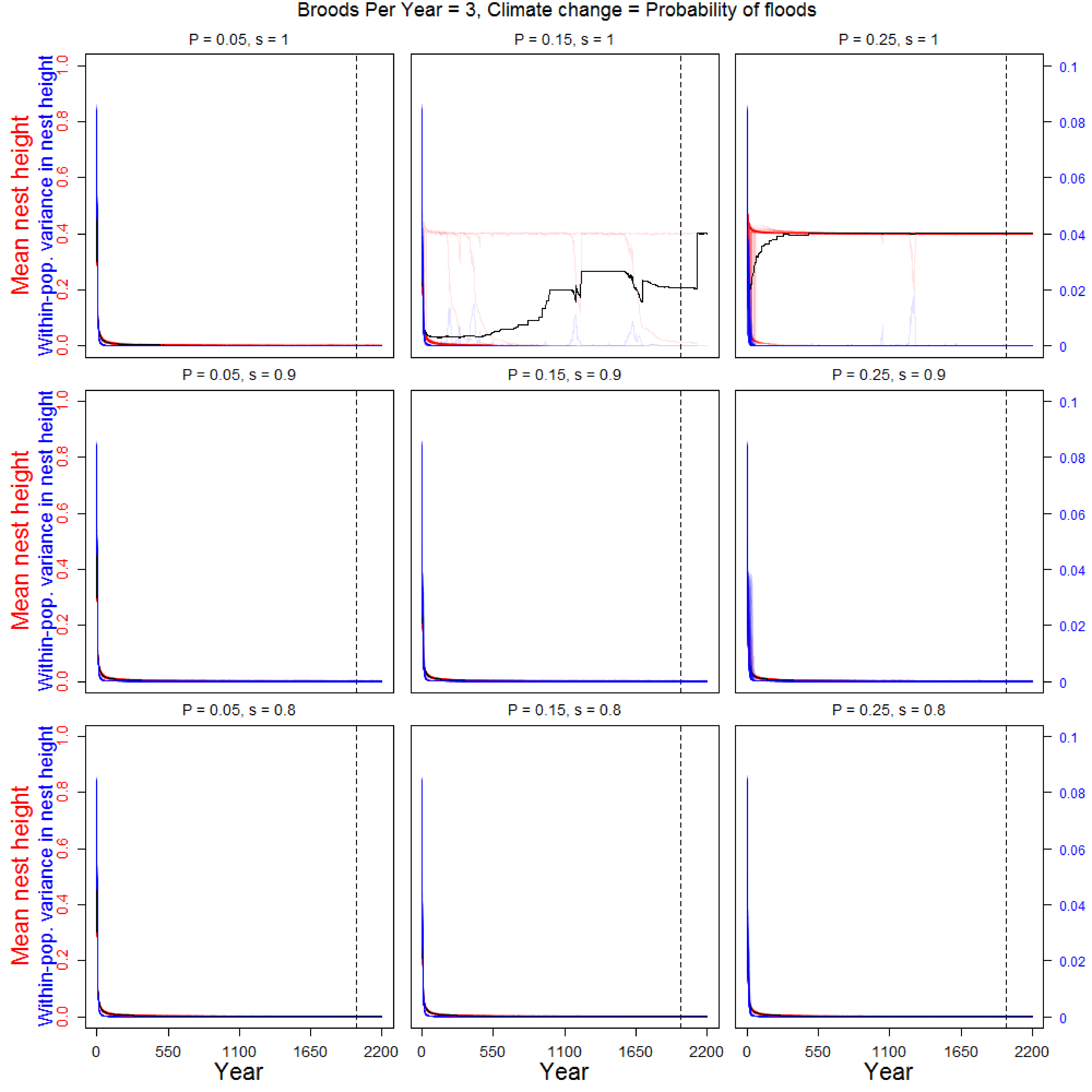
Broods Per Year = 3, Climate change = Probability of floods
P = 0.05, s = 1
P = 0.15, s = 1
P = 0.25, s = 1
P = 0.05, s = 0.9
P = 0.15, s = 0.9
P = 0.25, s = 0.9
P = 0.05, s = 0.8
P = 0.15, s = 0.8
P = 0.25, s = 0.8
Mean nest height
Within-pop. variance in nest height
0.0
0.2
0.4
0.6
0.8
1.0
0
0.02
0.04
0.06
0.08
0.1
0
550
1100
1650
2200
Year

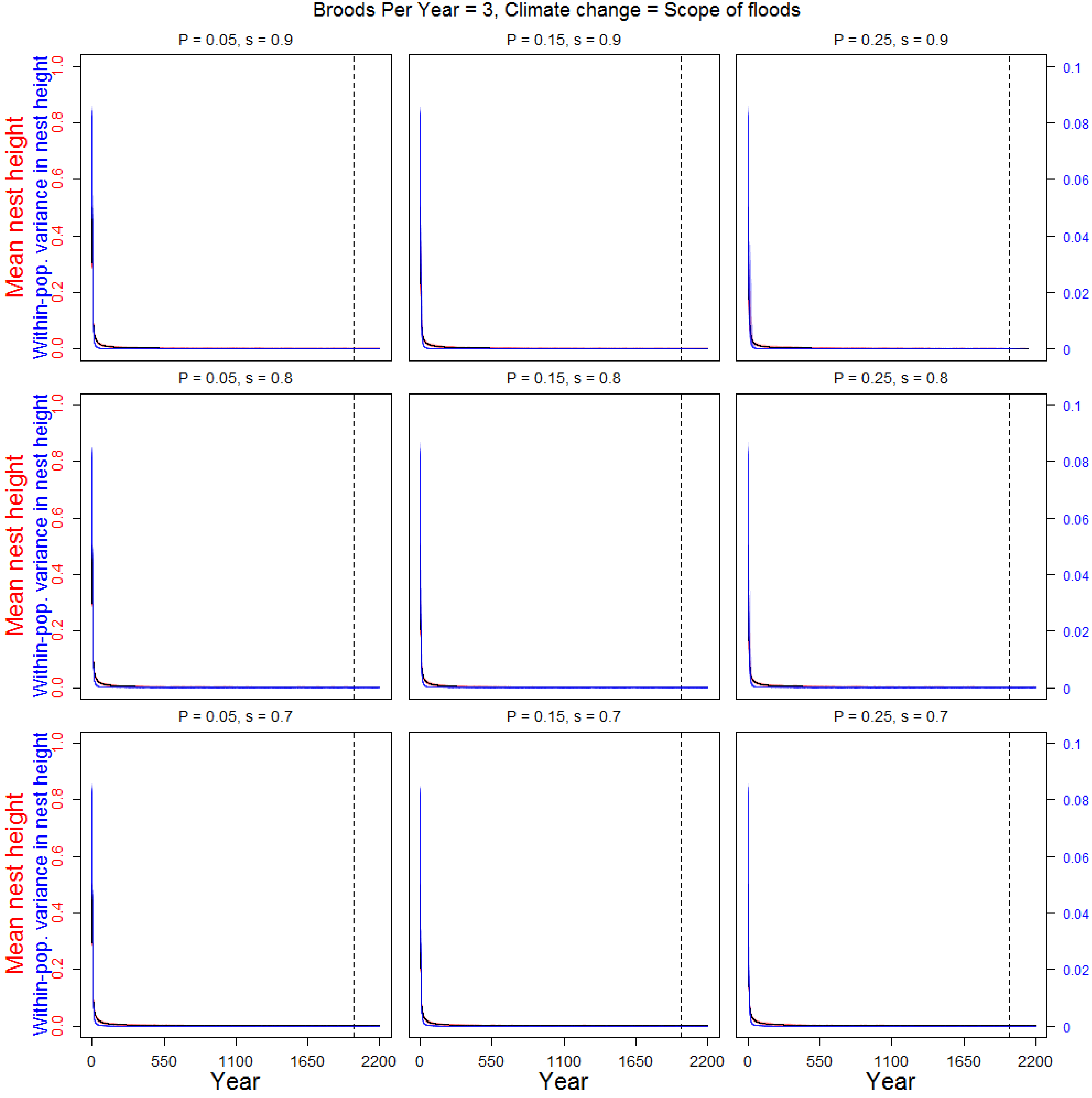

Broods Per Year = 3, Climate change = Scope of floods
P = 0.05, s = 0.9
P = 0.15, s = 0.9
P = 0.25, s = 0.9
P = 0.05, s = 0.8
P = 0.15, s = 0.8
P = 0.25, s = 0.8
P = 0.05, s = 0.7
P = 0.15, s = 0.7
P = 0.25, s = 0.7
Mean nest height
Within-pop. variance in nest height
0.0
0.2
0.4
0.6
0.8
1.0
0
0.02
0.04
0.06
0.08
0.1
0
550
1100
1650
2200
Year

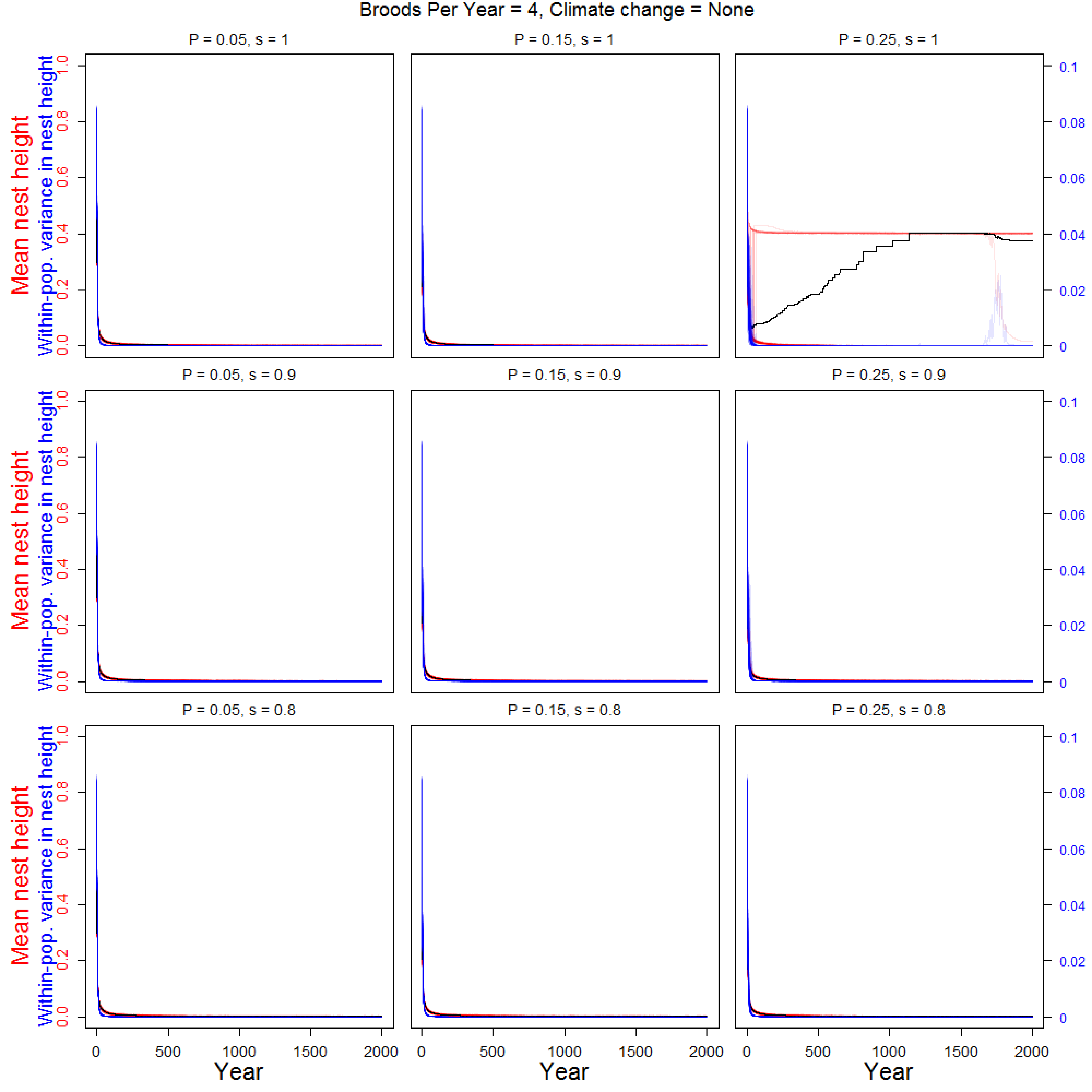
Broods Per Year = 4, Climate change = None
P = 0.05, s = 1
P = 0.15, s = 1
P = 0.25, s = 1
P = 0.05, s = 0.9
P = 0.15, s = 0.9
P = 0.25, s = 0.9
P = 0.05, s = 0.8
P = 0.15, s = 0.8
P = 0.25, s = 0.8
Mean nest height
Within-pop. variance in nest height
Year
0.0
0.2
0.4
0.6
0.8
1.0
0
0.02
0.04
0.06
0.08
0.1
0
500
1000
1500
2000

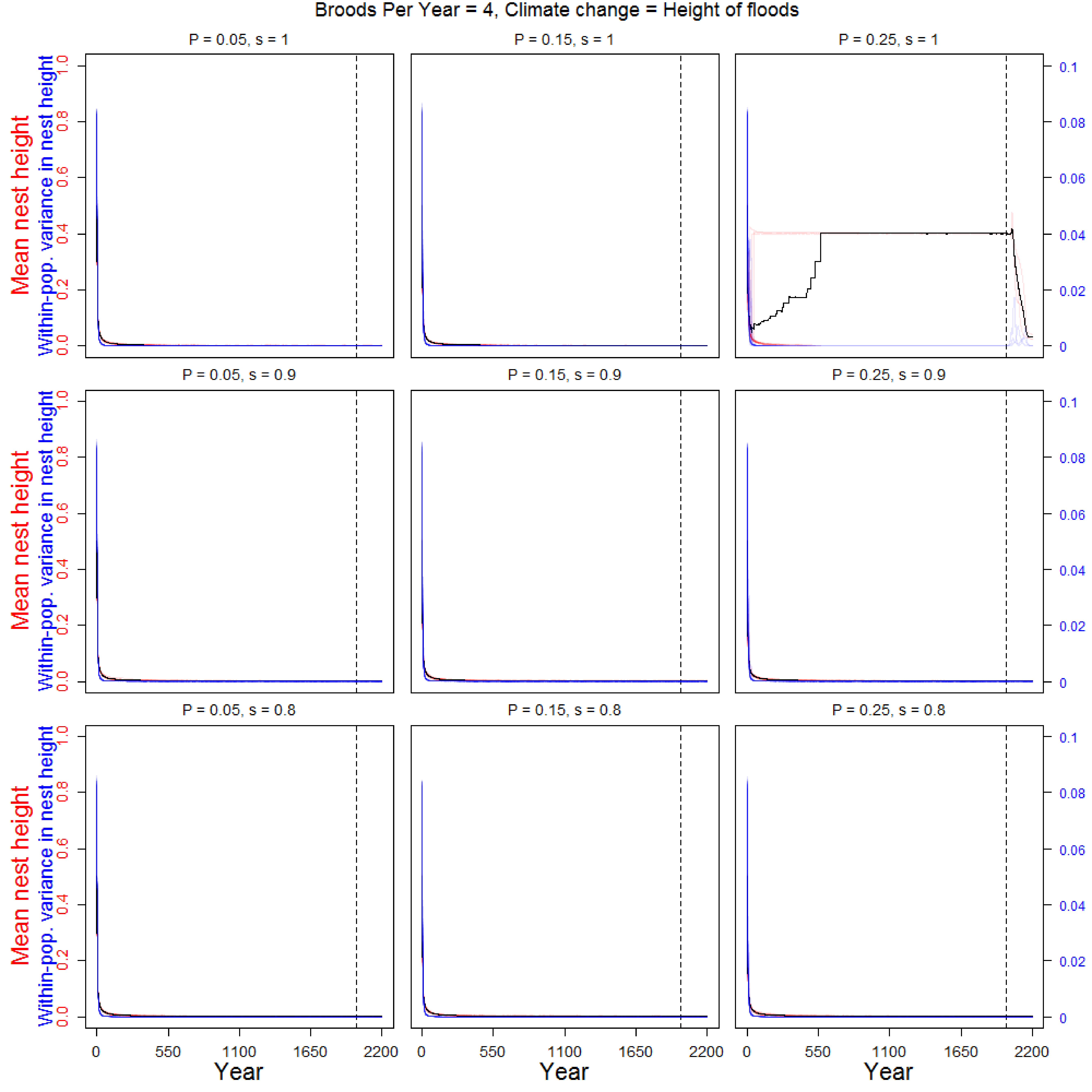

Broods Per Year = 4, Climate change = Height of floods
P = 0.05, s = 1
P = 0.15, s = 1
P = 0.25, s = 1
P = 0.05, s = 0.9
P = 0.15, s = 0.9
P = 0.25, s = 0.9
P = 0.05, s = 0.8
P = 0.15, s = 0.8
P = 0.25, s = 0.8
Mean nest height
Within-pop. variance in nest height
0.0
0.2
0.4
0.6
0.8
1.0
0
0.02
0.04
0.06
0.08
0.1
0
550
1100
1650
2200
Year

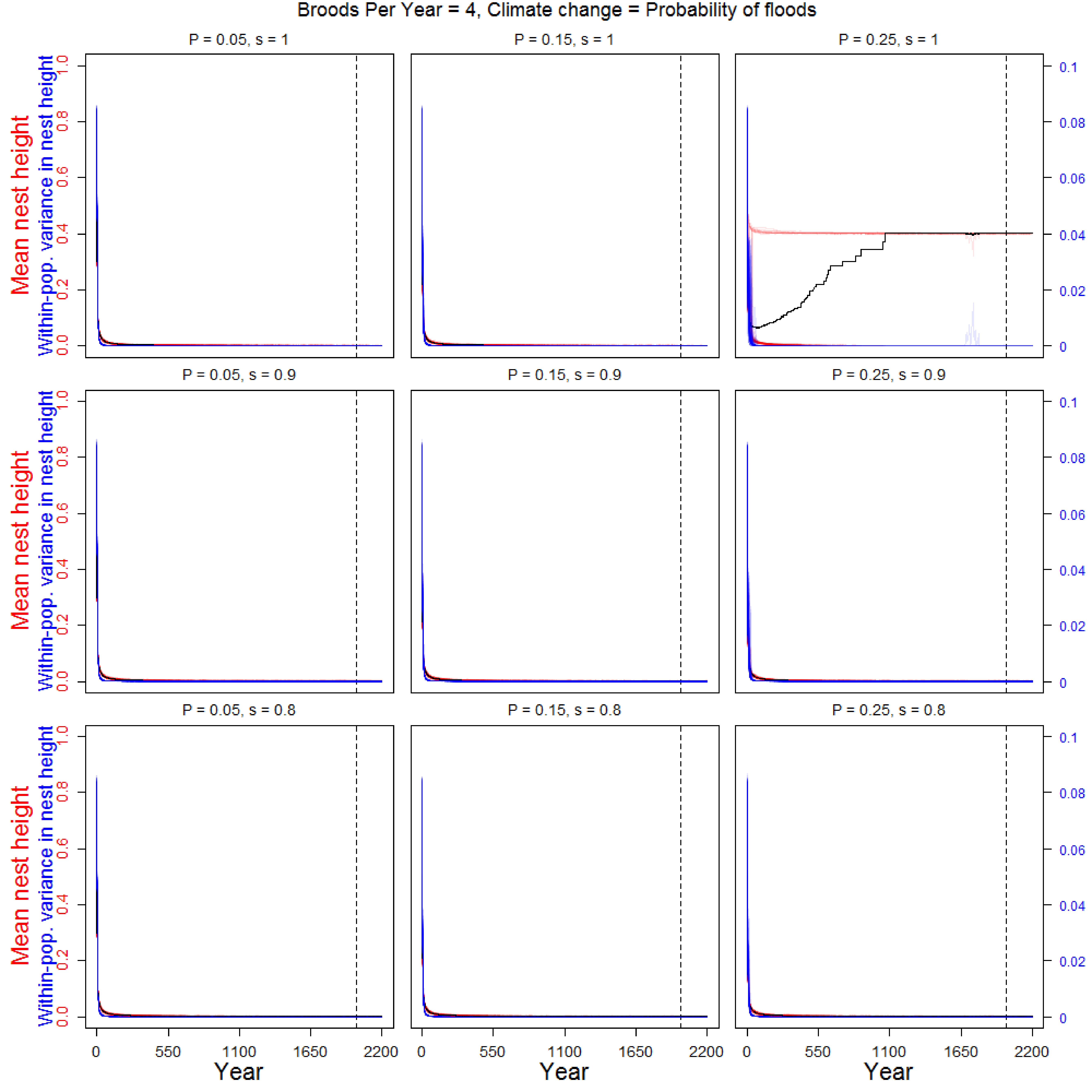

Broods Per Year = 4, Climate change = Probability of floods
P = 0.05, s = 1
P = 0.15, s = 1
P = 0.25, s = 1
P = 0.05, s = 0.9
P = 0.15, s = 0.9
P = 0.25, s = 0.9
P = 0.05, s = 0.8
P = 0.15, s = 0.8
P = 0.25, s = 0.8
Mean nest height
Within-pop. variance in nest height
0.0
0.2
0.4
0.6
0.8
1.0
0
0.02
0.04
0.06
0.08
0.1
0
550
1100
1650
2200
Year

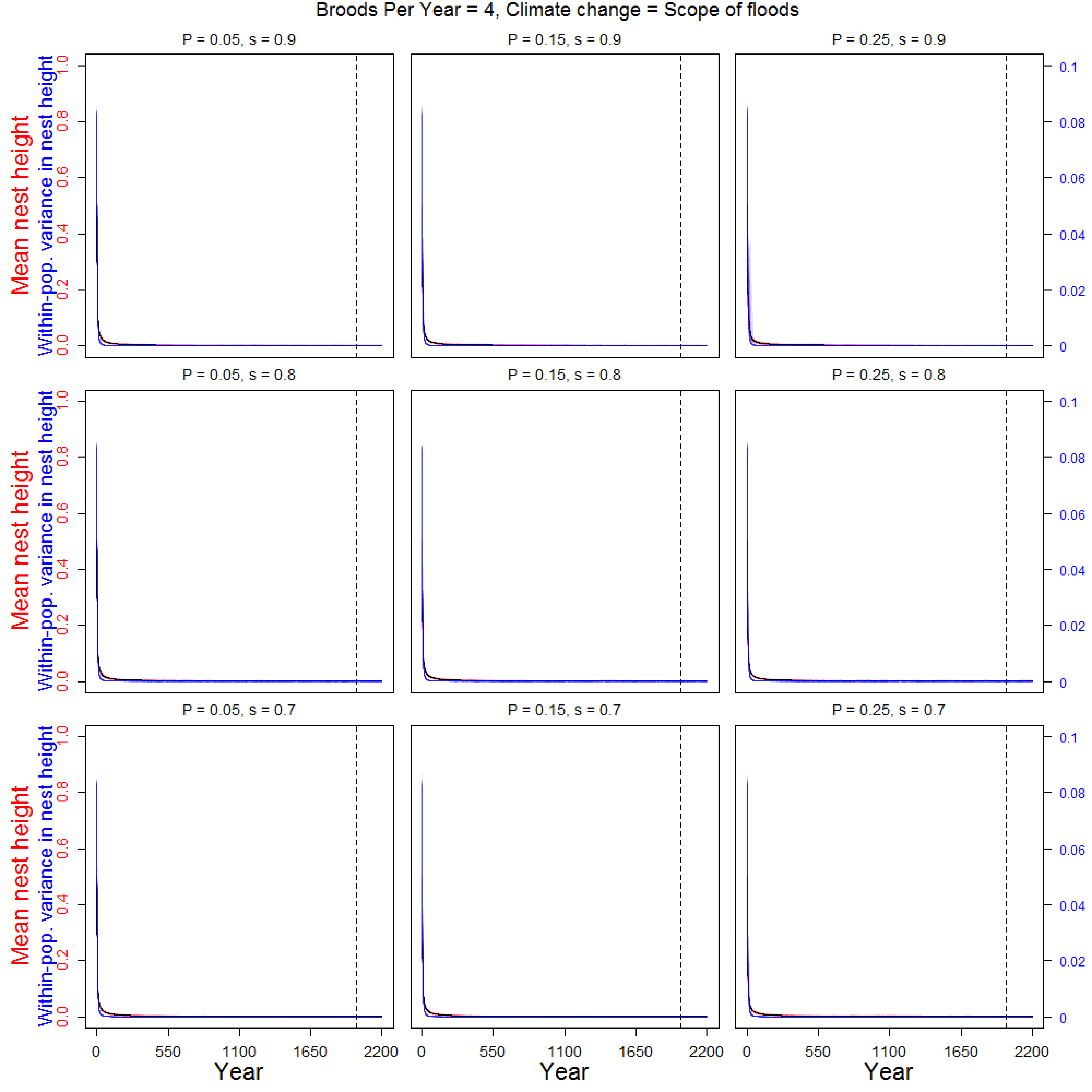

Broods Per Year = 4, Climate change = Scope of floods
P = 0.05, s = 0.9
P = 0.15, s = 0.9
P = 0.25, s = 0.9
P = 0.05, s = 0.8
P = 0.15, s = 0.8
P = 0.25, s = 0.8
P = 0.05, s = 0.7
P = 0.15, s = 0.7
P = 0.25, s = 0.7
Mean nest height
Within-pop. variance in nest height
0.0
0.2
0.4
0.6
0.8
1.0
0
0.02
0.04
0.06
0.08
0.1
0
550
1100
1650
2200
Year

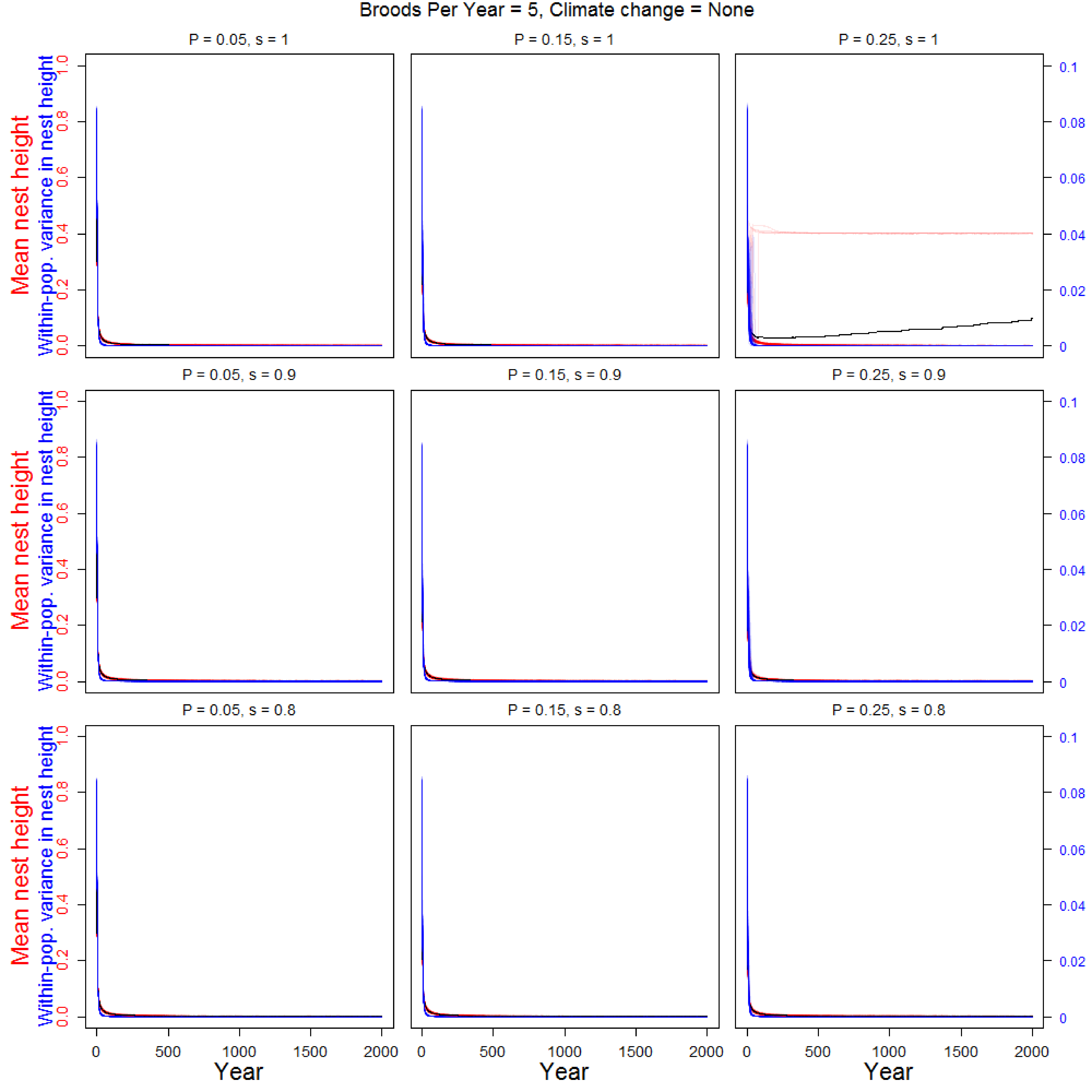

Broods Per Year = 5, Climate change = None
P = 0.05, s = 1
P = 0.15, s = 1
P = 0.25, s = 1
P = 0.05, s = 0.9
P = 0.15, s = 0.9
P = 0.25, s = 0.9
P = 0.05, s = 0.8
P = 0.15, s = 0.8
P = 0.25, s = 0.8
Mean nest height
Within-pop. variance in nest height
0.0
0.2
0.4
0.6
0.8
1.0
0
0.02
0.04
0.06
0.08
0.1
0
500
1000
1500
2000
Year

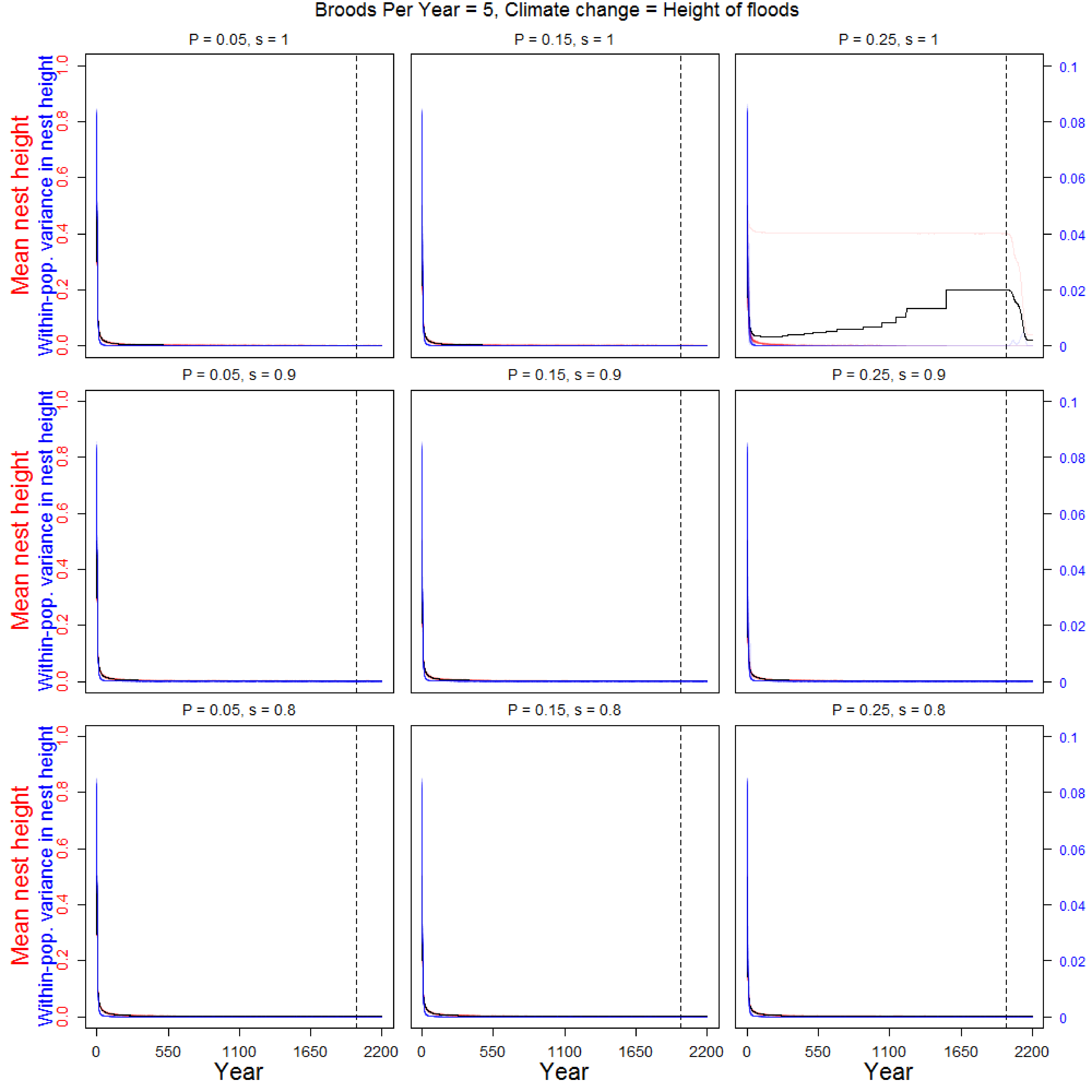

Broods Per Year = 5, Climate change = Height of floods
P = 0.05, s = 1
P = 0.15, s = 1
P = 0.25, s = 1
P = 0.05, s = 0.9
P = 0.15, s = 0.9
P = 0.25, s = 0.9
P = 0.05, s = 0.8
P = 0.15, s = 0.8
P = 0.25, s = 0.8
Mean nest height
Within-pop. variance in nest height
0.0
0.2
0.4
0.6
0.8
1.0
0
0.02
0.04
0.06
0.08
0.1
0
550
1100
1650
2200
Year

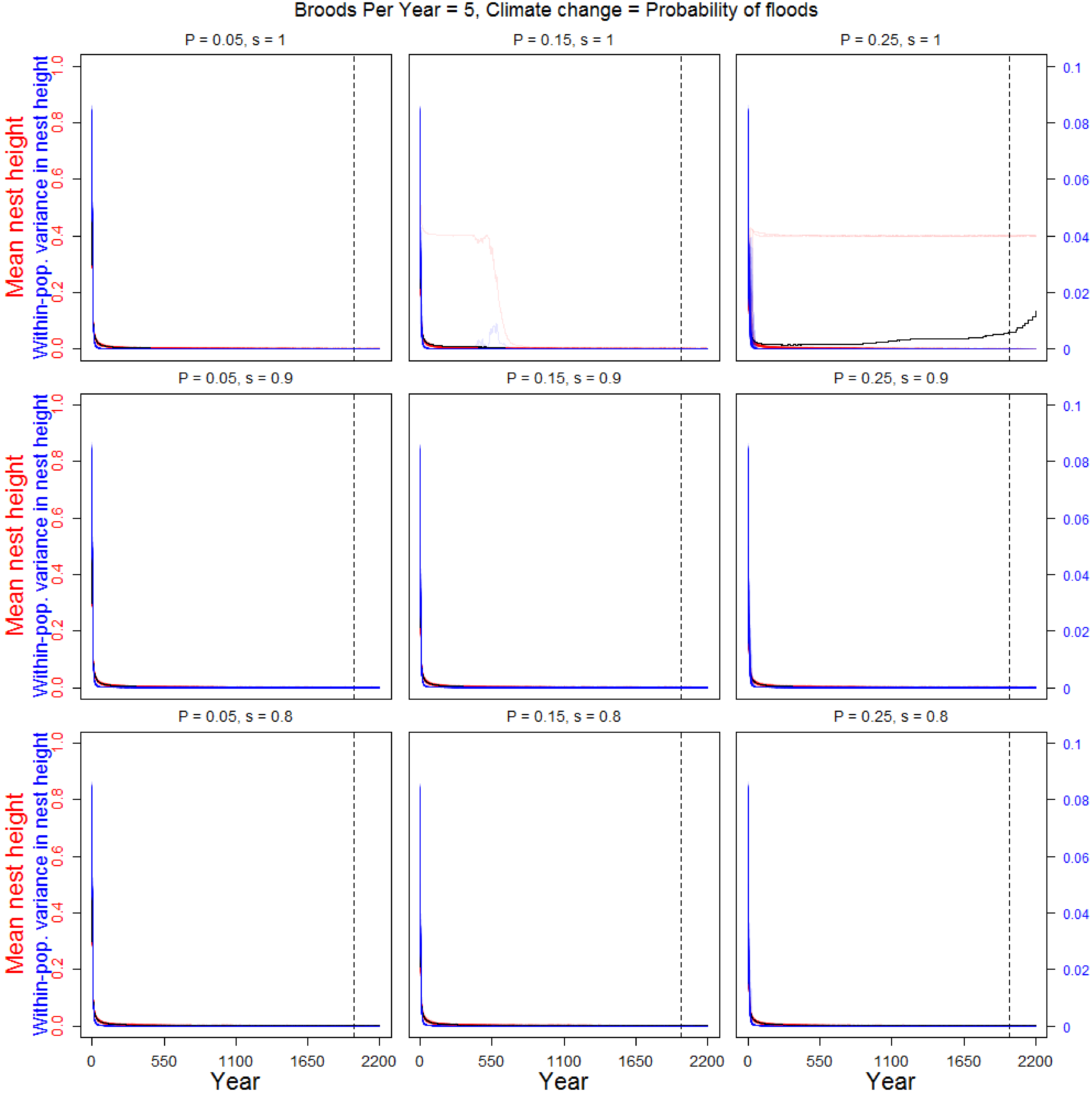

Broods Per Year = 5, Climate change = Probability of floods
P = 0.05, s = 1
P = 0.15, s = 1
P = 0.25, s = 1
P = 0.05, s = 0.9
P = 0.15, s = 0.9
P = 0.25, s = 0.9
P = 0.05, s = 0.8
P = 0.15, s = 0.8
P = 0.25, s = 0.8
Mean nest height
Within-pop. variance in nest height
0.0
0.2
0.4
0.6
0.8
1.0
0
0.02
0.04
0.06
0.08
0.1
0
550
1100
1650
2200
Year

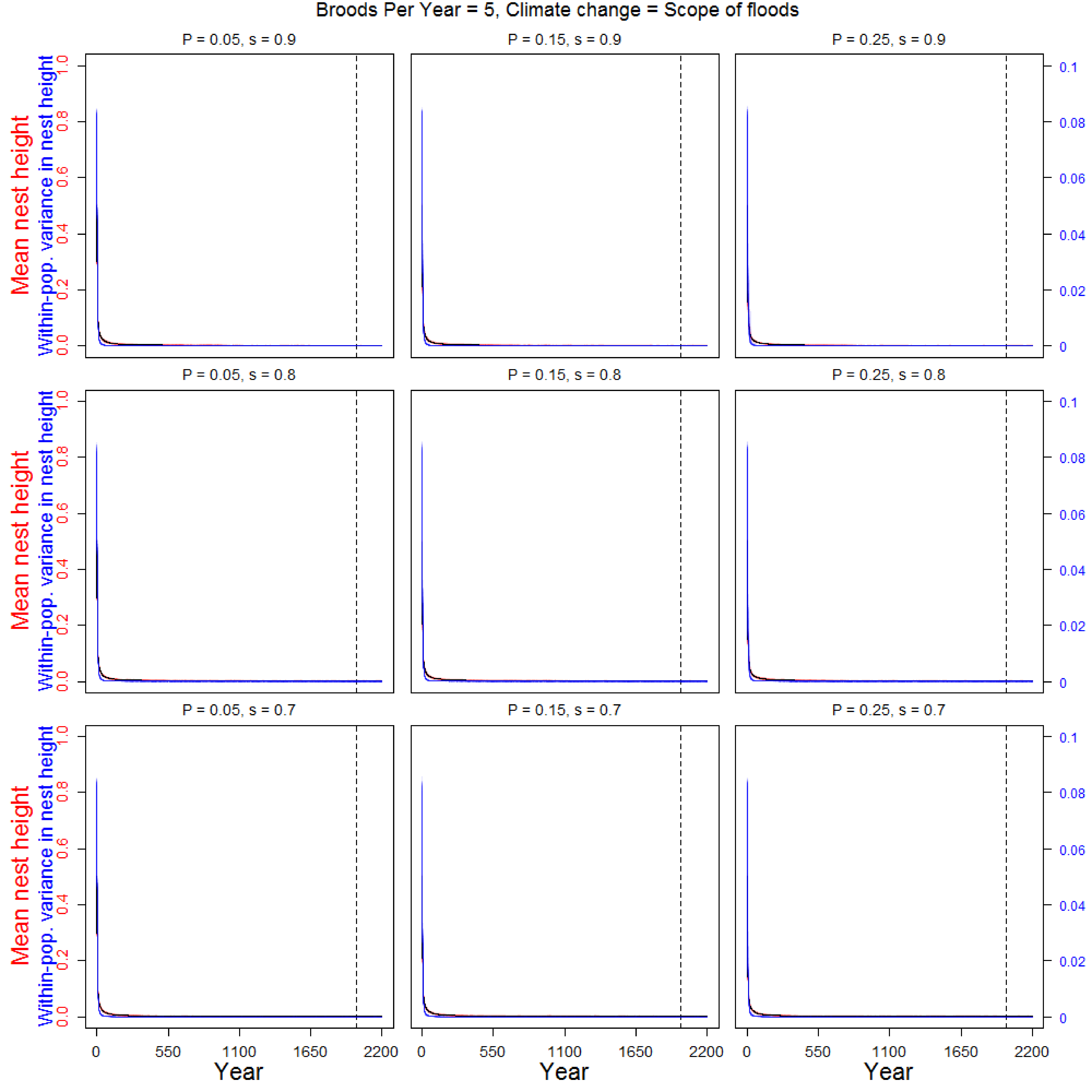

Broods Per Year = 5, Climate change = Scope of floods
P = 0.05, s = 0.9
P = 0.15, s = 0.9
P = 0.25, s = 0.9
P = 0.05, s = 0.8
P = 0.15, s = 0.8
P = 0.25, s = 0.8
P = 0.05, s = 0.7
P = 0.15, s = 0.7
P = 0.25, s = 0.7
Mean nest height
Within-pop. variance in nest height
0.0
0.2
0.4
0.6
0.8
1.0
0
0.02
0.04
0.06
0.08
0.1
0
550
1100
1650
2200
Year
